# WFS1 Deficiency Impairs PIAS4‐Associated SUMOylation and Increases Ubiquitin‐Mediated Spermatogenesis‐Related Protein Degradation Leading to Testicular Male Infertility

**DOI:** 10.1002/advs.78060

**Published:** 2026-10-04

**Authors:** Yunchuan Tian, Guicheng Zhao, Xiang Wang, Xinyao Tang, Gan Shen, Tiechao Ruan, Chuan Jiang, Shikun Zhao, Jincheng Zhang, Xinyue Cui, Liangchai Zhuo, Jun Ma, Yihong Yang, Tingting Lin, Ying Shen

**Affiliations:** ^1^ Department of Major Genetic Diseases, Key Laboratory of Birth Defects and Related Diseases of Women and Children (Sichuan University), Ministry of Education, West China Second University Hospital Sichuan University Chengdu China; ^2^ Reproductive Andrology and Sichuan Human Sperm Bank, Key Laboratory of Obstetric, Gynecologic and Pediatric Diseases and Birth Defects of Ministry of Education, West China Second University Hospital Sichuan University Chengdu China; ^3^ Reproduction Medical Center of West China Second University Hospital, Gynecologic and Pediatric Diseases and Birth Defects of Ministry of Education Sichuan University Chengdu China; ^4^ Chongqing Clinical Research Center for Reproductive Medicine Chongqing Health Center For Women and Children Chongqing China; ^5^ NHC Key Laboratory of Chronobiology Sichuan University Chengdu China

**Keywords:** fertilization failure, male infertility, mitochondrial sheath, SUMOylation, ubiquitination, WFS1

## Abstract

Although hundreds of genes have been shown to impair fertility in animal models, the identification of corresponding genetic causes in humans remains largely unknown. Here, we identified biallelic pathogenic variants in *WFS1* in three unrelated infertile men presenting with oligoasthenoteratozoospermia and fertilization failure. Remarkably, using the establishment of a *Wfs1* knockout (KO) mouse model, we found that WFS1 deficiency leads to disorganized sperm mitochondrial sheath assembly, structural abnormalities in the sperm head–neck junction, and defects in the sperm acrosome, which are consistent with the infertile phenotypes observed in affected individuals. Furthermore, meiotic defects were observed in spermatocytes from KO mice, particularly manifested as impaired sex chromosome synapsis. Mechanistically, WFS1 interacts with PIAS4 to promote the SUMOylation of key spermatogenesis‐associated proteins, including SUN5, HSF5, CFAP74, and IZUMO4, which in turn competitively inhibits their K48‐linked ubiquitin‐mediated degradation during spermatogenesis. Collectively, our study was the first to establish a critical role for WFS1 in human reproduction and to uncover the molecular mechanisms underlying WFS1‐mediated regulation of spermatogenesis. These findings provided both fundamental insights and important clinical implications for reproductive medicine.

## Graphical abstract

The findings establish a critical role for WFS1 in human male fertility. Mechanistically, WFS1 interacts with PIAS4 to promote the SUMOylation of key spermatogenesis‐associated proteins, which in turn competitively inhibits their K48‐linked ubiquitin‐mediated degradation during spermatogenesis.

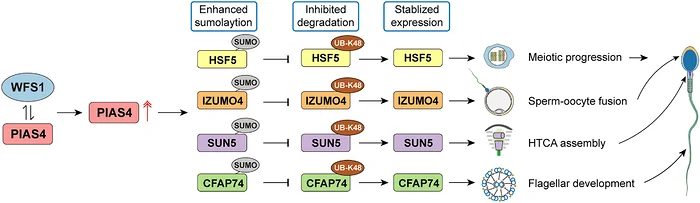

## 1 Introduction

Infertility has become an increasingly prominent public health concern worldwide, with male infertility accounting for nearly half of all cases [1]. Among its diverse etiologies, one of the most common phenotypes observed in infertile men is oligoasthenoteratozoospermia, which is characterized by low sperm count, poor motility, and abnormal morphology [2]. Genetic factors have been recognized as key contributors to male infertility, providing crucial insights into its underlying causes [3]. Although mutations in over 600 genes have been shown to impair fertility in animal models, the identification of corresponding genes in humans has progressed slowly [4]. Consequently, future research aimed at advancing our fundamental understanding of male infertility in humans is essential.

WFS1 (also known as Wolframin), an endoplasmic reticulum transmembrane protein, is widely expressed in various tissues and implicated in multiple cellular functions [5, 6]. Deficiency of WFS1 impairs endoplasmic reticulum homeostasis, consequently leading to cellular apoptosis [7, 8]. Moreover, pathogenic mutations in *WFS1* are classically associated with autosomal recessive Wolfram syndrome [OMIM (Mendelian Inheritance in Man) no. 222300], a rare progressive neurodegenerative disorder [9]. Previous studies have shown that some Wolfram syndrome patients also exhibit reduced reproductive capacity [5, 10, 11, 12, 13]. Intriguingly, *Wfs1* knockout (KO) mice have been reported to display not only features of Wolfram syndrome [14, 15] but also reduced male fertility [16]. However, the mechanisms by which WFS1 regulates spermatogenesis and its roles in human male reproduction remain limited.

In this study, we identified biallelic loss‐of‐function mutations in *WFS1* in three unrelated infertile men with oligoasthenoteratozoospermia through whole‐exome sequencing (WES). The corresponding mutant proteins exhibited significantly reduced expression. Clinically, these infertile patients predominantly presented with reduced sperm count, decreased sperm motility, and pronounced morphological abnormalities of the sperm head, neck, and flagellum. To further investigate the critical role of *Wfs1* in male reproduction, we generated *Wfs1* KO mice, which recapitulated the key reproductive phenotypes observed in the affected individuals. Intriguingly, we observed defective sex chromosome synapsis in KO mice by chromosome spread assays. In addition, impaired fertilization was also observed during in vitro fertilization (IVF) assays. Mechanistically, WFS1 interacts with the E3‐SUMO ligase PIAS4 to promote SUMOylation of key spermatogenesis‐associated proteins, including SUN5, HSF5, CFAP74, and IZUMO4, thereby competitively inhibiting K48‐linked ubiquitination and preventing their proteasomal degradation. Thus, WFS1 deficiency leads to downregulation of the E3‐SUMO ligase PIAS4, resulting in reduced SUMOylation and a concomitant increase in K48‐linked ubiquitination of key spermatogenesis‐associated proteins, ultimately causing male infertility. In summary, our study was the first to confirm that deleterious variants in *WFS1* cause human male infertility by disrupting spermatogenesis and fertilization and to elucidate the underlying pathological mechanisms, providing novel insights and potential strategies for the diagnosis and management of male infertility.

## 2 Materials and Methods

### 2.1 Study Participants and Sample Collection

This study enrolled 647 unrelated Chinese males diagnosed with oligoasthenoteratozoospermia at West China Second University Hospital and Chongqing Health Center for Women and Children. For the control group, testicular tissues were obtained from individuals with obstructive azoospermia who underwent diagnostic biopsies. In addition, sperm samples were collected from fertile Han Chinese men who had naturally fathered at least one child and fulfilled the criteria for normal semen parameters (sperm concentration ≥ 16 × 10^6^/mL and progressive motility ≥ 30%). All control donors participated voluntarily. The research protocol was reviewed and approved by the Ethics Committee of West China Second University Hospital (approval ID: 2023–160) and the Ethics Committee of Chongqing Health Center for Women and Children (approval ID: 2023–030). Written informed consent was obtained from every participant as well as from their family members prior to enrollment.

### 2.2 WES and Sanger Sequencing

Genomic DNA was isolated from peripheral blood samples of patients using the QIAamp DNA Blood Mini Kit (51126, QIAGEN). Exome enrichment was carried out with the SureSelect Human All Exon V6 Kit (G9706K, Agilent Technologies), followed by sequencing on the Illumina HiSeq X platform (15050091v07, Illumina). The mean sequencing depth across targeted exonic regions reached 100×, with 99.5% of bases covered at ≥20× depth. Variant annotation was performed using ANNOVAR, and common polymorphisms were filtered based on population databases, including the 1000 Genomes Project, gnomAD, and ExAC. After filtration, nonsynonymous single nucleotide variants were prioritized, and their functional consequences were assessed with prediction tools such as PolyPhen‐2, SIFT, SPIDEX, and CADD.

Candidate pathogenic variants detected by WES were further confirmed through Sanger sequencing, which also included parental DNA samples when available. PCR amplification was carried out using the ProFlex PCR System (A36962, Thermo Fisher), and the amplified products were sequenced on an ABI377A DNA sequencer (Applied Biosystems). The primer sequences used for amplification are provided in Table S1.

### 2.3 Papanicolaou Staining and Electron Microscopy

For Papanicolaou staining, semen smears were prepared on glass slides, air‐dried, and fixed in 95% (v/v) ethanol for 15 min. The slides were then sequentially processed through ethanol, Harris hematoxylin, acid ethanol, G‐6 orange, and EA‐50 green solutions. After staining, the preparations were dehydrated with graded ethanol series and mounted with neutral balsam for microscopic observation.

For scanning electron microscopy (SEM), approximately 200 spermatozoa from each sample were immobilized on 1‐cm diameter coverslips and fixed in 2.5% glutaraldehyde at 4°C for 4 h. The specimens were rinsed three times with 1× PBS and post‐fixed in 1% osmium tetroxide at 4°C for 1 h. Stepwise dehydration was performed with ethanol at 30%, 50%, 75%, 95%, and 100%, followed by drying with a CO_2_ critical‐point dryer (Eiko HCP‐2, Hitachi). The samples were mounted on aluminum stubs, coated with a thin gold layer using an ion sputter coater (Eiko E‐1020, Hitachi), and imaged under an SEM (S3400, Hitachi) at 10 kV accelerating voltage.

Transmission electron microscopy (TEM) was conducted using spermatozoa washed with Sperm Washing Medium. Samples were pre‐fixed in 3% glutaraldehyde and post‐fixed in 1% buffered osmium tetroxide. Following graded acetone dehydration, specimens were embedded in Epon 812 resin. Semi‐thin sections were first prepared for sperm localization under light microscopy, and ultrathin sections (40–70 nm) were subsequently cut. These sections were double‐stained with uranyl acetate and lead citrate, and examined with a TEM (TECNAI G2 F20, Philips) operating at 80 kV.

### 2.4 Real‐Time Quantitative PCR (RT‐qPCR)

Total RNA was isolated from samples using TRIzol reagent (15596026, Invitrogen). Reverse transcription was performed with 1 µg of RNA using the first Strand cDNA Synthesis Kit (HB210629, Yeasen) following the manufacturer's protocol.

Quantitative PCR was subsequently conducted with SYBR Green PCR Master Mix (B21202, Bimake) on a CFX Opus 96 Real‐Time PCR Detection System (Bio‐Rad). Primer sequences were designed using the Primer3 online program and are provided in Table S2.

### 2.5 Enzymatic Digestion of Testicular Tissues

Testicular tissues were obtained from adult C57BL/6 mice and patients with obstructive azoospermia to prepare single‐cell suspensions. Samples were washed in 1× PBS (10010023, Thermo Fisher Scientific), and seminiferous tubules were minced before transfer into a 15 mL tube containing 6 mL of primary digestion buffer (5.88 mL DMEM [11995073, Thermo Fisher Scientific], 120 µL collagenase [17100017, Sigma‐Aldrich], and 3 µL DNase I [D4263, Sigma‐Aldrich]). The suspension was incubated at 37°C for 10 min with gentle pipetting after 5 min. A secondary digestion buffer was freshly prepared, consisting of 5.28 mL DMEM, 600 µL trypsin‐EDTA (25200056, Thermo Fisher Scientific), 120 µL collagenase, and 3 µL DNase I, pre‐warmed to 37°C. After the first digestion, the mixture was allowed to settle for 2 min, the supernatant was discarded, and 6 mL of the secondary solution was added. The tissue suspension was gently pipetted 10 times and incubated at 37°C for an additional 12 min. Enzymatic activity was quenched with 2 mL fetal bovine serum (10270106, Gibco). The cell suspension was sequentially filtered through 100 and 40 µm strainers (22363547 and 22363546, Thermo Fisher Scientific). Cells were pelleted by centrifugation at 100 × g for 5 min, resuspended in DMEM containing 10% FBS, and examined under an inverted microscope to evaluate morphology and viability prior to downstream applications. All the animal experiments were performed in accordance with the recommendation of the Guide for the Care and Use of Laboratory Animals of the National Institutes of Health and the animal experiments were approved by the Experimental Animal Management and Ethics Committee of West China Second University Hospital.

### 2.6 Immunofluorescence Staining

Human and mouse germ cells were fixed with 4% paraformaldehyde and permeabilized using 0.3% Triton X‐100 for 10 min. To reduce nonspecific binding, the cells were blocked with 3% BSA for 1 h. Primary antibodies (listed in Table S3) were applied and incubated overnight at 4°C. On the following day, samples were rinsed three times with 1× PBS and then exposed to secondary antibodies for 1 h at room temperature. Nuclear staining was performed with DAPI (28718‐90‐3, Sigma‐Aldrich) for 10 min, and fluorescence images were acquired using a confocal laser scanning microscope (FV3000, Olympus).

Spermatocytes were distinguished from round spermatids based on their morphological characteristics. Spermatocytes are generally larger than round spermatids. SYCP3 immunostaining was used as an additional marker, as SYCP3 labels meiotic spermatocytes but is not detected in round spermatids. PNA staining served as the primary criterion for identification, as it is positive in spermatids (including round and elongating/elongated spermatids) but absent in spermatocytes.

For immunofluorescence analysis of mouse tissue, samples were initially fixed in 4% (w/v) paraformaldehyde, dehydrated through graded ethanol, embedded in paraffin, and sectioned at 5 µm thickness. After deparaffinization and rehydration, the sections were treated with 3% hydrogen peroxide and subjected to heat‐induced antigen retrieval in sodium citrate buffer. Following blocking with goat serum, sections were incubated overnight at 4°C with primary antibodies (listed in Table S3). On the next day, sections were treated with secondary antibodies and counterstained with DAPI. Images were obtained with a fluorescence microscope (Axio, Zeiss).

### 2.7 Western Blot Analysis

Proteins were extracted from cells and mouse testes tissues using a universal lysis buffer (HR0259, Biotek) supplemented with a protease inhibitor cocktail (B14012, Bimake). Protein concentration was quantified with the Pierce BCA Protein Assay Kit (23225, Thermo Fisher Scientific). Equal amounts of denatured protein were separated by 10% SDS–PAGE and transferred onto PVDF membranes (IPVH00010, Millipore). Membranes were blocked in 5% skim milk prepared in TBST for 1 h at room temperature, followed by overnight incubation with primary antibodies at 4°C (Table S3). After three washes with TBST, membranes were incubated with HRP‐conjugated secondary antibodies for 1 h at room temperature (Table S3). Immunoreactive bands were detected using enhanced chemiluminescence reagent (32209, Thermo Fisher Scientific) and visualized on photographic film.

### 2.8 Generation of the *Wfs1* KO Mouse Model

All animal procedures were approved by the Experimental Animal Management and Ethics Committee of West China Second University Hospital, Sichuan University (No. 20230150) and were performed in accordance with institutional guidelines. A *Wfs1* KO mouse model was established using the CRISPR–Cas9 system. Briefly, Cas9 mRNA, *Wfs1* signal‐guide RNA1 (5’‐AGGGAAGACGTCCACTCTTAAGG‐3’), and *Wfs1* signal‐guide RNA2 (5’‐ACCTTGTTGATCTGTTGATCTGG‐3’) were co‐injected into zygotes. Exon 3 of the *Wfs1*‐201 transcript (ENSMUST00000043964.13), an 83 bp coding region essential for protein function, was selected as the target region. Following microinjection, embryos were cultured and subsequently transferred into the oviducts of pseudopregnant females at 0.5 days post‐coitum. Genomic mutations in founder mice and their offspring were confirmed by PCR (Table S2).

### 2.9 Fertility Assessment

Adult male *Wfs1* KO mice and their WT littermates (n = 5 per group) were each housed with two WT females for 6 months. Vaginal plug formation was monitored daily in the morning as an indicator of successful mating. Females with confirmed plugs were separated and housed individually, while new WT females were introduced to maintain the mating ratio. The total number of litters produced and the number of pups per litter were recorded throughout the breeding period.

### 2.10 Proteomic Analysis

Testicular tissues from 8‐week‐old mice were prepared for liquid chromatography–tandem mass spectrometry (LC–MS/MS) analysis using a standardized procedure. Briefly, four volumes of lysis buffer (8 M urea and 1% protease inhibitor cocktail) were added to the testicular tissue powder, followed by sonication for 3 min on ice using a high‐intensity ultrasonic processor (Scientz). The remaining debris was removed by centrifugation at 12 000 × g for 10 min at 4°C. The supernatant was collected, and protein concentration was determined using a BCA assay kit according to the manufacturer's instructions. Proteins were precipitated by slowly adding TCA to a final concentration of 20% (w/v), followed by incubation for 2 h at 4°C. The precipitated proteins were collected by centrifugation at 4500 × g for 5 min at 4°C, washed three times with pre‐cooled acetone, and air‐dried briefly. The protein pellets were redissolved in 200 mM TEAB and ultrasonically dispersed. The samples were reduced with 5 mM dithiothreitol for 30 min at 56°C and alkylated with 11 mM iodoacetamide for 15 min at room temperature in the dark. Subsequently, trypsin was added at a 1:50 trypsin‐to‐protein mass ratio for overnight digestion. The resulting peptides were desalted using a Strata X SPE column. The obtained data were annotated using Gene Ontology (GO, http://ebi.ac.uk/GOA/), InterProScan for protein domains, and KEGG pathway analysis via the KAAS mapper tool. Protein–protein interaction networks were predicted using STRING (https://string‐db.org/).

### 2.11 In Vitro Fertilization (IVF)

Four‐week‐old C57BL/6J female mice were superovulated by injecting 5 IU of pregnant mare serum gonadotropin (PMSG) followed by 5 IU of human chorionic gonadotropin (hCG) 48 h later. Sperm was released from the cauda epididymis of 8‐week‐old male mice, and sperm capacitation was performed for 50 min using test yolk buffer with HEPES (TYH) solution. Cumulus‐oocyte complexes (COCs) were obtained from the ampulla of the uterine tube 14 h after hCG injection. The ampulla was torn with a syringe needle, and the COCs were gently squeezed onto liquid drops of human fallopian tube fluid (HTF) medium. The COCs were then incubated with ∼5 µl of the sperm suspension in HTF liquid drops at 37°C under 5% CO_2_. After 6 h, the eggs were washed several times using HTF medium to remove cumulus cells and then transferred to liquid drops of potassium simplex optimized medium (KSOM).

### 2.12 Intracytoplasmic Sperm Injection (ICSI)

ICSI was performed following standard protocols. Female KM mice (4 weeks old) were superovulated by intraperitoneal injection of 5 IU equine chorionic gonadotropin (eCG; ProSpec, HOR‐272), followed 48 h later by 5 IU human chorionic gonadotropin (hCG) to obtain metaphase II (MII) oocytes. Collected oocytes were maintained in Chatot–Ziomek–Bavister medium (Easycheck, M2750) at 37.5°C under 5% CO_2_.

Spermatozoa were isolated from the cauda epididymis and incubated in human tubal fluid (HTF; Easycheck, M1150). To separate sperm heads, samples were subjected to repeated freeze–thaw cycles. Individual sperm heads were then microinjected into MII oocytes using a Piezo‐assisted micromanipulation system mounted on a NIKON inverted microscope (PrimeTech, Osaka, Japan). The injections were performed in Whitten's‐HEPES medium supplemented with polyvinyl alcohol (Gibco, 12360‐038) and cytochalasin B (3.5 µg/mL; Sigma‐Aldrich, C‐6762). Following injection, oocytes were cultured in G‐Plus medium (Vitrolife, 10132) at 37.5°C in a 5% CO_2_ atmosphere. All animal procedures were approved by the Experimental Animal Management and Ethics Committee of West China Second University Hospital, Sichuan University.

### 2.13 Statistical Analysis

Statistical analyses were conducted using GraphPad Prism (version 8.4.0; GraphPad Software, Boston, MA, USA) and SPSS (version 18.0; IBM Corp., Armonk, NY, USA). Data are presented as mean ± standard error of the mean (SEM). Comparisons between two groups were performed using an unpaired, two‐tailed Student's t‐test assuming normal distribution. A *p* value < 0.05 was considered indicative of statistical significance.

## 3 Result

### 3.1 Identification of bi‐allelic Variants in *WFS1* in Chinese Infertile Men with Oligoasthenoteratozoospermia and Fertilization Failure

In this study, a cohort of 647 unrelated Chinese infertile men diagnosed with oligoasthenoteratozoospermia was recruited to investigate the genetic etiology through WES. Among these patients, we identified three unrelated individuals (P68, P219, and P553) harboring five missense variants in *WFS1* (NM_006005.3; NP_005996.2) from three families (Figure 1A). Specifically, patient 68 carried a compound heterozygous missense variant of c.1817T>G (p.V606G) and c.2200C>T (p.L734F); patient 219 possessed a homozygous missense variant, c.2315G>A (p.R772H); and patient 553 harbored a compound heterozygous missense variant of c.763G>A (p.A255T) and c.2122C>T (p.R708C) (Figure 1A). All variants were subsequently confirmed by Sanger sequencing. Moreover, these identified variants in *WFS1* were extremely rare or absent in population databases, including the ExAC Browser, 1000 Genomes Project, and gnomAD, and were predicted to be deleterious by in silico bioinformatic tools such as SIFT, M‐CAP, and PolyPhen‐2 (Table 1). Notably, these variants were located in exons 7 and 8, and the affected amino acid residues are highly conserved among diverse vertebrate species (Figure 1B).

**FIGURE 1:**
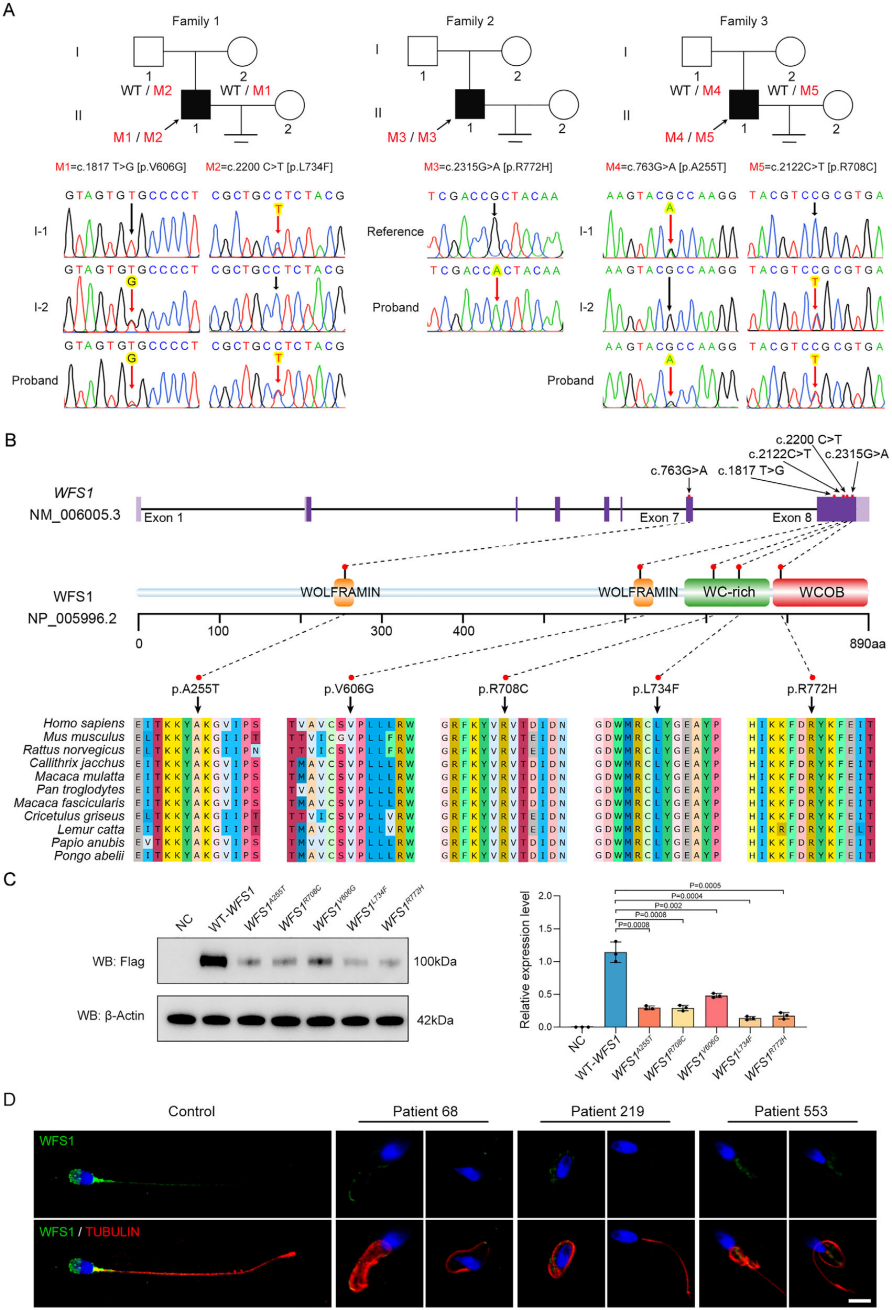
Identification of *WFS1* variants in three unrelated infertile men. (A) Family pedigrees. The black square denotes the probands. Sanger sequencing was performed to validate the presence of candidate pathogenic variants in affected individuals and unaffected family members. Mutated alleles in *WFS1* are marked with red arrows, whereas black arrows indicate reference alleles. (B) Genomic and protein‐level localization of the variant sites. The black arrows highlight the positions of the variants within the genomic and protein structure of *WFS1*. The affected amino acid residues are highly conserved across multiple vertebrate species. (C) Western blot analysis of HEK293T cells transfected with WT and Mut *WFS1* overexpression plasmids. (D) Immunofluorescence staining of WFS1 in sperm from controls and patients. Red: α‐Tubulin; green: WFS1; blue: DAPI. Scale bars, 5 µm.

**TABLE 1:** Semen and variant analysis in the *WFS1‐*mutated patients.

		Patient 68		Patient 219	Patient 553		Reference
	Age	34		37	27		
**Semen parameters**	Semen concentration (10^6^ /ml)	8.2		1.8	2.4		≥16
	Progressive motility (%)	13		17	12		≥30
	Normal sperm morphology (%)	1.4		0.5	1.2		≥4
**Variant**	cDNA mutation ^a^	c.1817 T>G	c.2200 C>T	c.2315G>A	c.763G>A	c.2122C>T	
	Protein changes	p.V606G	p.L734F	p.R772H	p.A255T	p.R708C	
	Mutation type	Missense	Missense	Missense	Missense	Missense	
	Genotype	Compound heterozygous		Homozygous	Compound heterozygous		
**Allele frequency**	ExACBrowser	0.000041	0.000087	0.000017	N/A	N/A	
	GnomAD‐All	0.000035	0.000016	0.000020	0.000006	0.000081	
	1000 Genomes Project	N/A ^b^	N/A	N/A	N/A	N/A	
**Function prediction**	SIFT	Deleterious	Deleterious	Deleterious	Tolerated	Deleterious	
	PolyPhen‐2	Possibly damaging	Possibly damaging	Benign	Possibly damaging	Probably damaging	
	M‐CAP	Probably damaging	Probably damaging	Probably damaging	Probably damaging	Probably damaging	
	CADD ^C^	23	24.4	23.3	23.5	28.4	
	ACMG	PM2, PM5 ^d^	PM2, PP3 ^e^	PM1, PM2	PM1, PM2	PM2, PP3	

^a^ NM_006005.3.

^b^ Abbreviation: N/A, not available.

^c^ CADD score > 4 is predicted to be damaging.

^d^ PM: Pathogenic Moderate, indicating moderate pathogenic evidence in ACMG classification.

^e^ PP: Pathogenic Supporting, indicating supporting pathogenic evidence in ACMG classification.

To evaluate the impact of these candidate variants on protein structure, we performed in silico three‐dimensional modeling. The analysis revealed that all five variants, located on α‐helices and β‐sheets, disrupted hydrogen bonding or altered the interatomic distances between neighboring amino acids, which is likely to compromise protein stability and function (Figure S1A). Furthermore, we constructed wild‐type (WT) and five mutant (Mut) *WFS1* plasmids and transfected them into HEK293 cells, and observed a markedly reduced expression of the mutant proteins compared with WT (Figure 1C). Consistently, Western blot analysis of peripheral blood samples from the three patients showed reduced WFS1 protein levels compared with controls (Figure S1B). Immunofluorescence assays further showed that WFS1 localized to the sperm acrosome and the midpiece of the sperm flagellum in control samples, whereas sperm from the three patients displayed substantially reduced fluorescence intensity (Figure 1D). Collectively, these pathogenic variants in *WFS1* potentially contributed to the genetic etiology of human male infertility.

### 3.2 Severe Sperm Decapitation Defects, Head Malformations, and Mitochondrial Disorganization in the Patients with bi‐allelic *WFS1* Variants

Routine semen analysis of the three patients harboring bi‐allelic variants in *WFS1* revealed reduced count, decreased motility, and nearly universal abnormal morphology of the sperm (Table 1). Subsequent sperm morphology assessment using Papanicolaou staining revealed pronounced head abnormalities, including tapered, pyriform, round, and irregular forms, as well as neck defects manifested as bent‐neck and decapitated spermatozoa, accompanied by flagellar abnormalities (Figure 2A). Transmission electron microscopy (TEM) further revealed evident acrosomal defects accompanied by chromatin decondensation in sperm heads (Figure 2B). Similarly, peanut agglutinin (PNA) staining of sperm acrosomes from the patients was also abnormal (Figure S3A). TEM analysis also showed disorganization of the head‐to‐tail coupling apparatus (HTCA) in the sperm neck region (Figure 2C). In addition, the sperm flagella primarily exhibited disorganized mitochondrial sheaths (Figure 2D), accompanied by disruptions of the typical “9 + 2” axonemal arrangement, including missing central‐pair microtubules (CPs), irregular outer dense fibers (ODFs), and defective microtubule doublets (MTDs) (Figure S2). Moreover, Patient 68 was diagnosed with diabetes, a typical feature of Wolfram syndrome, but denied other typical manifestations such as optic atrophy, deafness, diabetes insipidus, or psychiatric disorders. Similarly, patients 219 and 553, due to cultural and personal reasons, believed they did not present any of these characteristic symptoms and declined further examinations. Taken together, these results demonstrated that deleterious biallelic variants in *WFS1* may contribute to oligoasthenoteratozoospermia in affected individuals.

**FIGURE 2:**
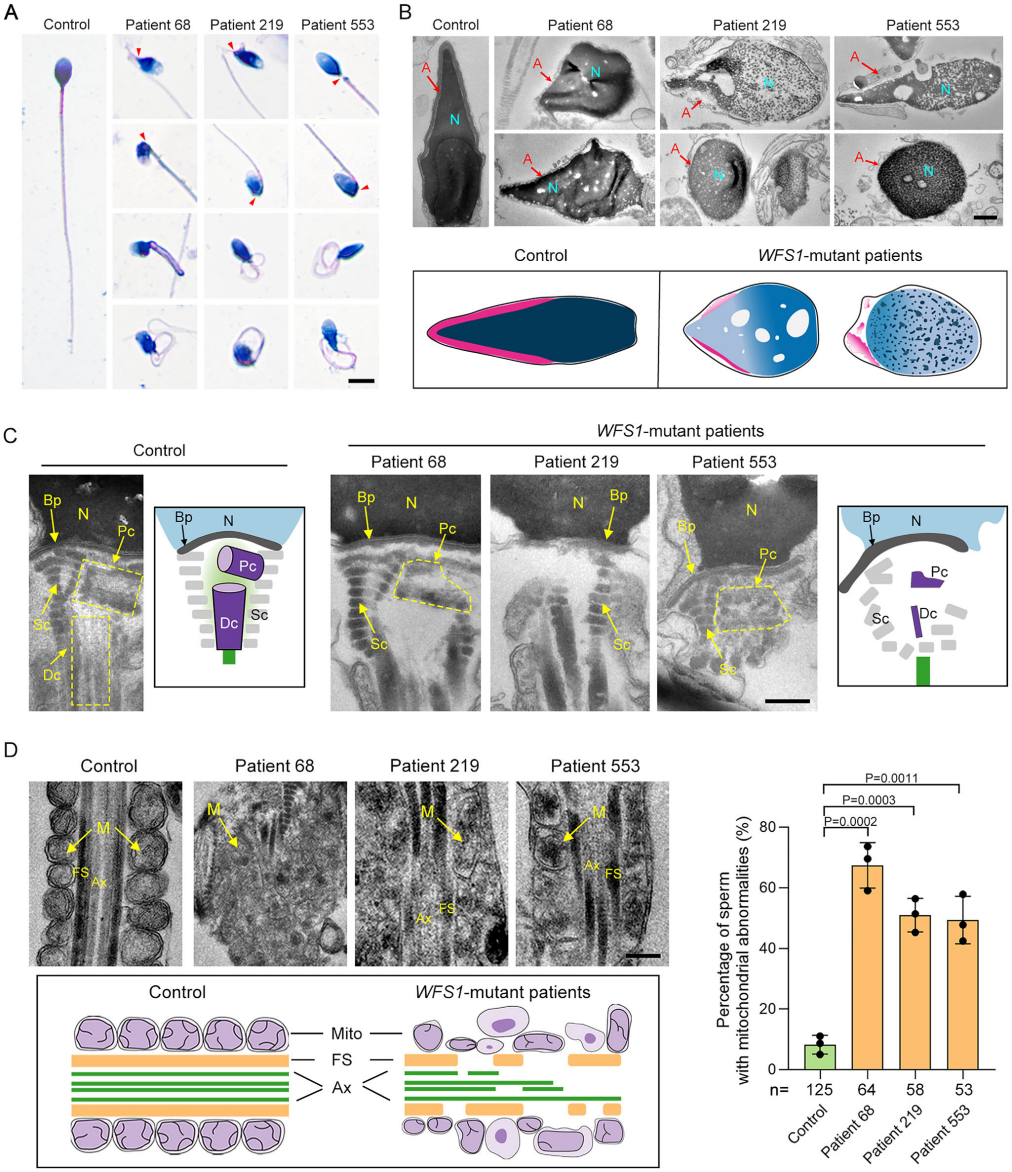
Morphological and ultrastructural abnormalities in sperm from patients with *WFS1* variants. (A) Papanicolaou staining revealed abnormal sperm morphology in patients’ samples compared with the control group. Red arrowheads indicated abnormalities in the sperm neck. Scale bars, 5 µm. (B) TEM analysis showing ultrastructural defects in the sperm head of patients, including chromatin decondensation and acrosomal abnormalities. A, acrosome; N, nucleus. Scale bars, 500 nm. A schematic diagram of abnormal sperm head morphology is shown below. (C) TEM analysis showing ultrastructural defects in the HTCA in the sperm neck of patients. A schematic diagram is shown on the right. Bp, basal plate; Sc, segmented column; Pc, proximal centriole; Dc, distal centriole; N, nucleus. Scale bars, 250 nm. (D) TEM analysis showing ultrastructural defects in the mitochondria of the sperm midpiece in patients. Representative images are shown, with a schematic diagram below and quantification presented on the right. Scale bar: 250 nm. *n* indicates the total number of sperm mitochondrial sheaths analyzed across three independent technical replicates from the patient sample. Two‐sided Student's *t*‐test. The *P* values are indicated in the graphs. Mito, mitochondria; FS, fibrous sheath; Ax, axoneme.

### 3.3 *Wfs1* KO Mice Exhibit Disrupted Testicular Architecture and Impaired Sex Chromosome Synapsis

WFS1 was expressed across multiple stages of mouse and human spermatogenesis and showed colocalization with PNA in spermatids and with SYCP3 in spermatocytes (Figure S4A–C). Further co‐immunofluorescence staining of testicular sections for WFS1 and SOX9 (a marker of Sertoli cells) confirmed that WFS1 is also expressed in Sertoli cells (Figure S4D). To further investigate its functional role, we sought to establish an in vivo model. Given that the three affected patients collectively carried five distinct *WFS1* variants, and the corresponding mutant WFS1 protein was markedly downregulated, generating a knock‐in model to recapitulate all variants was deemed impractical. Therefore, we chose to generate *Wfs1* KO mice using CRISPR–Cas9 technology to investigate the functional role of *Wfs1* in spermatogenesis (Figure 3A). The successful establishment of the KO model was confirmed by PCR (Figure S5A), western blot (Figure S5D), RT‐qPCR (Figure S5E), and immunofluorescence in testicular tissue and sperm (Figure S5F,G). Mating tests revealed that the KO male mice were completely infertile, whereas the KO females retained fertility (Figure 3B). In addition, KO mice also exhibited marked growth retardation, characterized by reduced body size and decreased body weight, consistent with a previous study (Figure S5B) [17]. However, the testis‐to‐body weight and epididymis‐to‐body weight ratios showed no significant difference compared with WT mice (Figure S5C).

**FIGURE 3:**
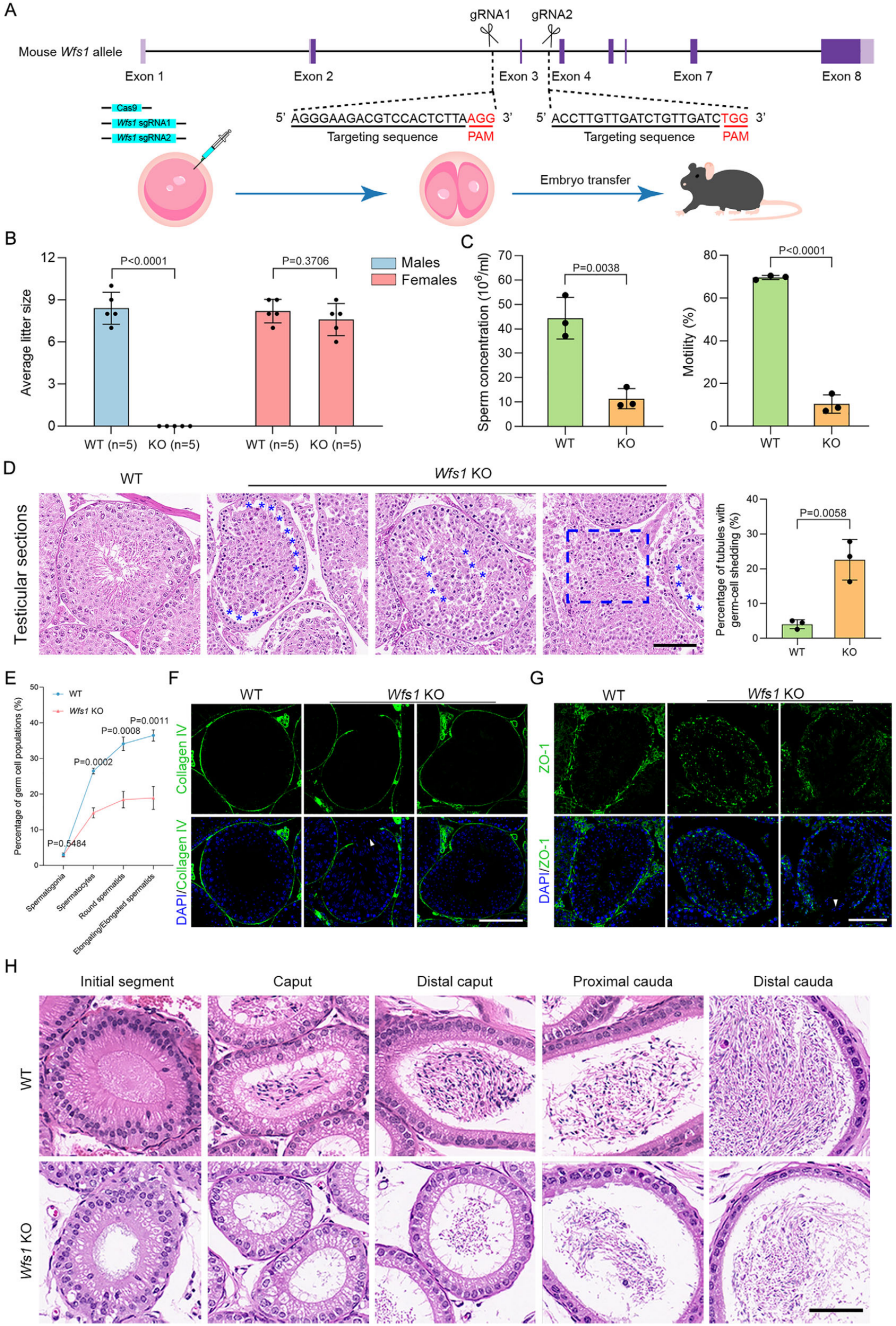
*Wfs1* deficiency leads to male infertility and impaired spermatogenesis in mice. (A) Schematic diagram of the CRISPR/Cas9 targeting strategy used to generate *Wfs1* KO mice. Exon 3 of *Wfs1* was targeted. (B) Fertility assessment of WT and KO mice. KO males were completely sterile, whereas KO females exhibited normal fertility (*n*, number of mice analyzed; two‐sided Student's *t* test; error bars, mean ± SEM; the *P* values are indicated in the graphs). (C) Sperm concentration and motility were significantly reduced in *Wfs1* KO mice compared with control groups, as assessed by CASA. Data are presented as mean ± SEM from three groups. The *P* values are indicated in the graphs. Statistical significance was observed among all groups (*p* < 0.05). (D) H&E staining of testicular sections from adult male *Wfs1* KO mice showing germ cell shedding into the lumen and interstitium (N = 3 biologically independent WT and KO mice; scale bars, 125 µm). Blue stars indicate disruption of the stratified layers of spermatogenic cells and sloughing of germ cells. Blue dashed boxes highlight the detachment of germ cells into the interstitial space due to disruption of the seminiferous epithelium. Quantification of the percentage of seminiferous tubules exhibiting germ cell shedding is shown on the right (two‐sided Student's t‐test; error bars, mean ± SEM; *p* values are indicated in the graphs). (E) Quantification of the proportions of different germ cell populations in testes from WT and *Wfs1* KO mice. The proportions of spermatocytes, round spermatids, and elongating/elongated spermatids were decreased in *Wfs1* KO testes. Percentages were calculated using the total germ cell number in WT testes as the reference denominator for both genotypes. (n  =  3 biologically independent WT and KO mice; two‐sided Student's t test; error bars, mean ± SEM). *P* values are indicated in the graphs. (F) Immunostaining for Collagen IV in testes from WT and *Wfs1* KO mice (green, Collagen IV; blue, DAPI; scale bars, 125 µm). White arrows indicate regions of germ cell detachment into the interstitial space. (G) Immunostaining for ZO‐1 in testes from WT and *Wfs1* KO mice (green, ZO‐1; blue, DAPI; scale bars, 125 µm). White arrows indicate regions of germ cell detachment into the interstitial space. (H) H&E staining of epididymal sections showing reduced sperm counts in the lumen of *Wfs1* KO mice compared with WT controls (N = 3 biologically independent WT and KO mice; scale bars, 50 µm).

We further tested serum testosterone and FSH levels in *Wfs1* KO males (9.23 ± 1.83 nmol/L and 4.93 ± 0.45 mIU/mL, respectively) and found them to be comparable to those in WT controls (10.8 ± 1.65 nmol/L and 4.87 ± 0.49 mIU/mL, respectively), showing no significant differences, which is consistent with the previous report [16]. To further elucidate whether the infertility in *Wfs1* KO male mice originates from testicular defects, we performed hematoxylin and eosin (H&E) staining of mouse testes. *Wfs1* KO testes exhibited increased germ cell shedding, characterized by the detachment of spermatocytes, round spermatids, and elongating/elongated spermatids into the tubular lumen or interstitial space (Figure 3D). The identity of these shedding germ cells was further confirmed by immunofluorescence staining (Figure S6A,B). In addition, we performed immunostaining for Collagen IV, a marker of the tubular basement membrane, and ZO‐1, a marker of the blood–testis barrier. In WT testes, Collagen IV showed continuous staining encircling the seminiferous tubular basement membrane, whereas discontinuous Collagen IV staining patterns were detected in regions exhibiting germ cell detachment into the interstitial space (Figure 3F), indicating compromised basement membrane integrity. Moreover, the proportion of seminiferous tubules exhibiting fragmented ZO‐1 staining patterns was increased in KO testes (Figure 3G). In contrast, WT testes displayed continuous ZO‐1 signals surrounding Sertoli cells along the seminiferous tubules, consistent with preserved blood–testis barrier integrity. Subsequently, we conducted immunofluorescence staining of testicular tissue for SOX9 (Figure S7A), c‐Kit (a marker of spermatogonia) (Figure S7B), SYCP3 (a marker of spermatocytes) (Figure S7C), and PNA (a marker of the acrosome) (Figure S7D), revealing a reduction in spermatocytes and spermatids, whereas the numbers of spermatogonia and Sertoli cells showed no significant differences (Figure 3E). Furthermore, mature sperm counts in the epididymal lumen of KO males were markedly reduced compared with WT controls, providing initial evidence for impaired spermatogenesis (Figure 3H).

We further performed chromosome spread assays to examine whether *Wfs1* KO spermatocytes exhibited meiotic abnormalities. The result of co‐immunostaining for SYCP3 and γH2AX revealed no defects in the initiation and early processing of programmed double‐strand breaks (DSBs) during the leptotene/zygotene stages, followed by normal sex body formation at the pachytene stage (Figure S8). Moreover, we subsequently assessed synapsis by co‐immunostaining for SYCP3 and SYCP1. In WT spermatocytes, SYCP1 began to colocalize with SYCP3 during synapsis initiation at the zygotene stage and became fully colocalized at pachytene, except in the non‐pseudoautosomal region (non‐PAR) of sex chromosomes (Figure 4A). In contrast, the proportion of spermatocytes exhibiting abnormal sex chromosome synapsis was significantly increased in *Wfs1* KO mice, characterized by absent SYCP1 signals within the PAR or aberrant SYCP1 localization outside the PAR (Figure 4B). These defects were further substantiated by HORMAD1 staining, which marks unsynapsed chromosome axes. In WT pachytene spermatocytes, HORMAD1 signals were restricted to non‐PARs, whereas in *Wfs1* KO spermatocytes, aberrant HORMAD1 localization was also observed in PARs (Figure 4C). We next performed immunostaining for MLH3, a marker of meiotic crossover sites, and observed an increased proportion of *Wfs1* KO spermatocytes with absent MLH3 foci within the PAR region of the XY chromosomes (Figure 4D). Furthermore, no significant differences were observed in the leptotene and zygotene stages, whereas the proportion of pachytene spermatocytes was mildly increased and that of diplotene spermatocytes was significantly decreased in *Wfs1* KO testes (Figure 4E).

**FIGURE 4:**
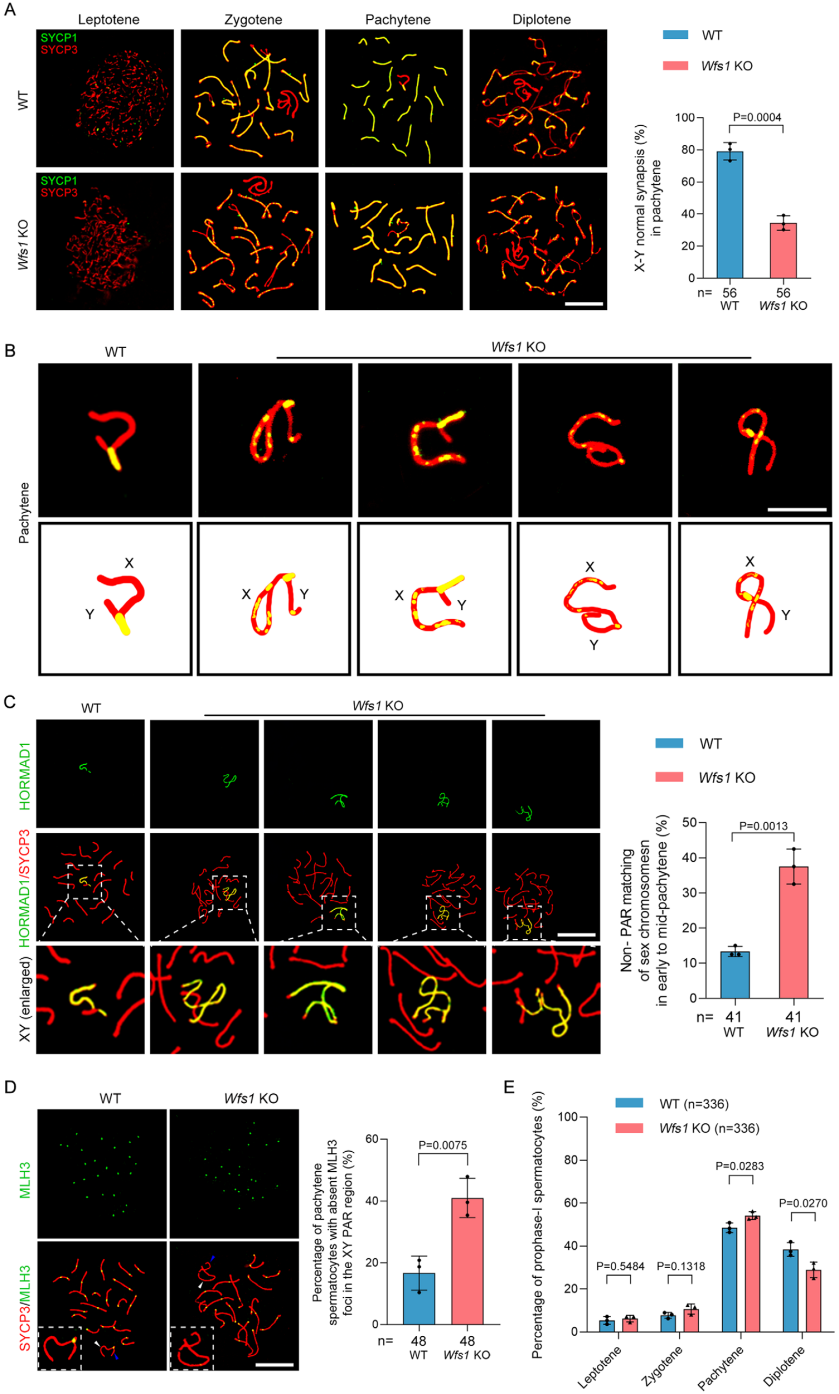
Defective sex chromosome synapsis in *Wfs1* KO male mice. (A) Immunostaining of surface‐spread spermatocytes from WT and *Wfs1* KO mice with SYCP3 and SYCP1. *Wfs1* KO mice exhibited abnormal sex chromosome synapsis. Quantification of normal X–Y synapsis per nucleus in pachytene‐stage spermatocytes is shown (N = 3 biologically independent WT and KO mice; *n*, the total number of nuclei analyzed; two‐sided Student's *t* test; *P* values are indicated in the graphs; error bars, mean ± SEM; green: SYCP1; red: SYCP3; scale bars, 10 µm). (B) The enlarged panels exhibited abnormally synapsed sex chromosomes (green: SYCP1; red: SYCP3; scale bars, 5 µm). (C) Chromosome spreads of WT and *Wfs1* KO spermatocytes stained for SYCP3 and HORMAD1. High‐magnification images of the XY chromosomes are shown below. Quantification of non‐PAR X–Y associations per nucleus in pachytene spermatocytes is shown (N = 3 biologically independent WT and KO mice; *n*, the total number of nuclei analyzed; two‐sided Student's *t* test; *P* values are indicated in the graphs; error bars, mean ± SEM; green: HORMAD1; red: SYCP3; scale bars, 10 µm). (D) Chromosome spreads of WT and *Wfs1* KO spermatocytes stained for SYCP3 and MLH3. High‐magnification images of the XY chromosomes are shown below. Quantification of the percentage of pachytene spermatocytes lacking MLH3 foci in the XY PAR region is shown (N = 3 biologically independent WT and KO mice; *n*, the total number of nuclei analyzed; two‐sided Student's *t* test; *P* values are indicated in the graphs; error bars, mean ± SEM; green: MLH3; red: SYCP3; scale bars, 10 µm). The white arrow indicates the X chromosome, and the blue arrow indicates the Y chromosome. (E) Percentages of substages of spermatocytes in *Wfs1* KO mice. Quantification of the proportion of spermatocytes (N = 3 biologically independent WT mice and KO mice; n, the total number of nuclei analyzed; two‐sided Student's t test; *P* values are indicated in the graphs; error bars, mean ± SEM).

### 3.4 Severe Defects in Sperm Head and Mitochondrial Sheaths in *Wfs1* KO Mice

To further assess sperm quality, we performed computer‐assisted sperm analysis (CASA), which revealed that *Wfs1* KO males exhibited reduced motility and decreased sperm counts (Figure 3C and Table 2). Notably, Papanicolaou staining revealed that *Wfs1* KO mice exhibited a markedly increased proportion of spermatozoa with head and neck abnormalities compared with WT mice (Figure 5A,B). Consistently, SEM further confirmed pronounced head and neck abnormalities (Figure 5C) and disrupted mitochondrial organization in the flagellar midpiece of *Wfs1* KO sperm (Figure 5D). Ultrastructural analysis further demonstrated that *Wfs1* KO spermatozoa exhibited evident acrosomal defects (Figure 5E). Notably, we found that chromatin decondensation was less severe and occurred in a smaller proportion of *Wfs1* KO spermatozoa than in patient spermatozoa (Figure 5E), and also observed structural abnormalities in the sperm neck region (Figure 5F). Moreover, flagellar ultrastructure was severely disrupted, primarily characterized by disorganized mitochondrial sheaths (Figure 5G), accompanied by irregular ODFs and defective MTDs (Figure S9A). Consistent with these findings, immunofluorescence staining for TOM20, a mitochondrial marker, further confirmed mitochondrial abnormalities in both mouse and human sperm (Figure S3B,C). Taken together, these findings established WFS1 as an essential regulator of spermatogenesis, whose deficiency led to male infertility in mice by disrupting germ cell development and causing profound defects in sperm morphology, motility, and ultrastructure.

**TABLE 2:** Semen analysis by CASA in *Wfs1* KO mice.

	WT	*Wfs1* KO	*P* ^**b**^ value
**Semen parameters**			
Sperm concentration (10^6^/ml) ^a^, ^b^	44.43 ± 8.53	11.43 ± 4.14	0.003
Motility (%) ^b^	69.60 ± 0.98	10.40 ± 4.31	<0.001
Progressive motility (%) ^b^	60.23 ± 10.02	7.10 ± 2.35	<0.001
**Sperm locomotion parameters**			
Curvilinear velocity (VCL) (µm/s) ^b^	68.80 ± 7.60	16.63 ± 2.65	<0.001
Straight‐line velocity (VSL) (µm/s) ^b^	34.07 ± 2.75	5.06 ± 0.97	<0.001
Average path velocity (VAP) (µm/s) ^b^	46.63 ± 5.95	7.70 ± 1.15	<0.001
Amplitude of lateral head displacement (ALH) (µm) ^b^	1.26 ± 0.12	0.35 ± 0.11	<0.001
Linearity (LIN) ^b^	0.49 ± 0.02	0.31 ± 0.06	0.006
Wobble (WOB = VAP/VCL) ^b^	0.68 ± 0.04	0.47 ± 0.07	0.011
Straightness (STR = VSL/VAP)	0.73 ± 0.07	0.65 ± 0.03	0.155
Beat‐cross frequency (BCF) (Hz) ^b^	5.43 ± 1.17	0.52 ± 0.19	0.002

^a^ Epididymides and vas deferens.

^b^ A significant difference, two‐sided Student's *t*‐test. n = 3 biologically independent wild‐type (WT) mice or knockout (KO) mice.

**FIGURE 5:**
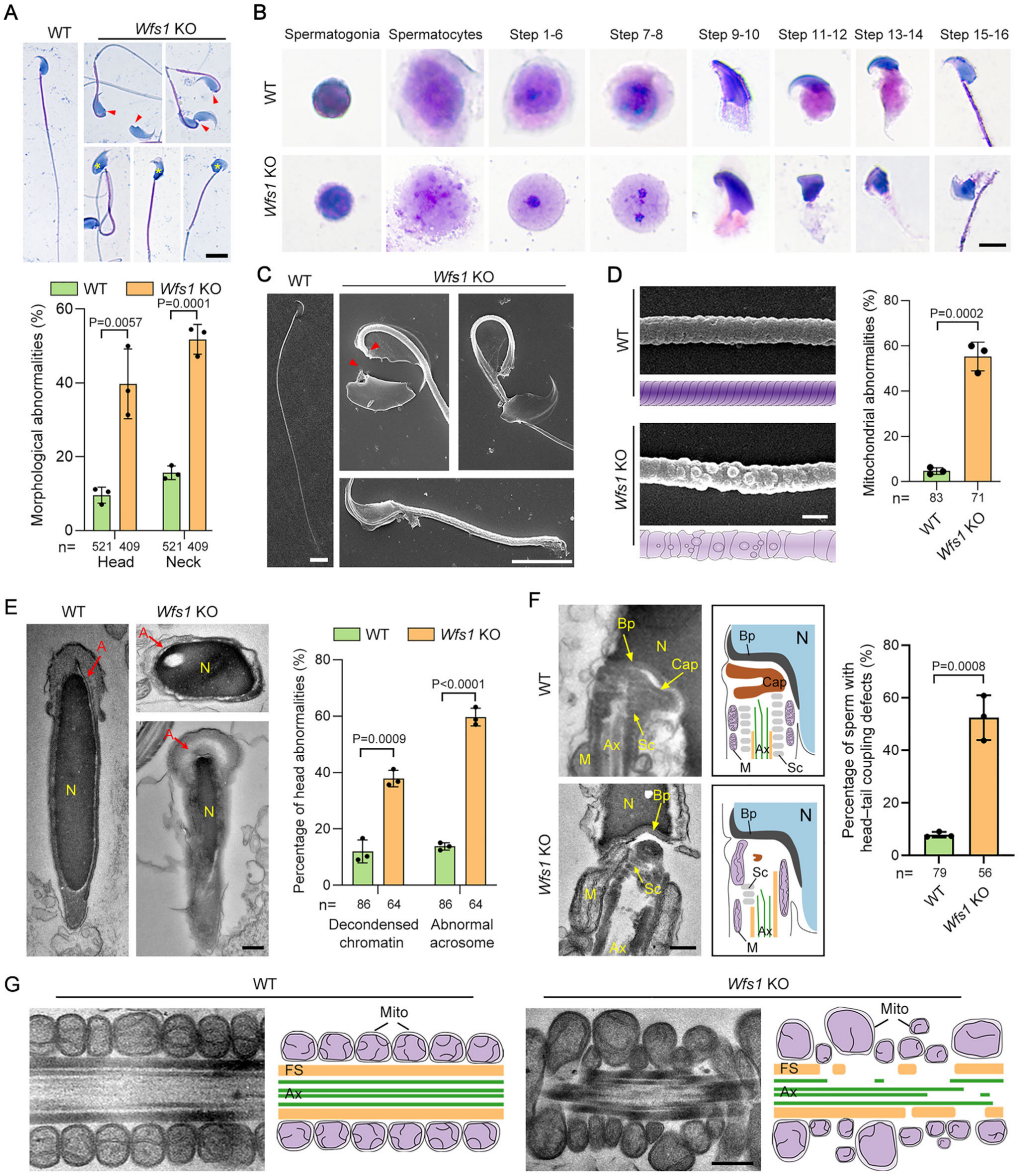
*Wfs1* deficiency leads to severe sperm morphological and ultrastructural abnormalities in mice. (A) Abnormal sperm morphology observed in *Wfs1* KO mice by Papanicolaou staining, compared with WT controls. Red arrowheads indicated abnormalities in the sperm neck, and yellow stars indicated defects in the sperm head. Scale bars, 5 µm. Quantification of sperm head and neck abnormalities is shown below. N = 3 biologically independent mice; *n* indicates the number of sperm analyzed. Two‐sided Student's t‐test; error bars, mean ± SEM; *P* values are indicated in the graphs. (B) Papanicolaou staining showing abnormal sperm morphology at different stages of spermatogenesis in *Wfs1* KO mice (scale bars, 5 µm). (C) SEM showing abnormal sperm morphology in *Wfs1* KO mice. Red arrowheads indicate abnormalities in the sperm neck. Scale bars, 5 µm. (D) SEM analysis revealing disorganized mitochondrial arrangement in the sperm midpiece of *Wfs1* KO mice. A schematic diagram is shown below, and quantification is presented on the right. Scale bars, 500 nm. N = 3 biologically independent mice; *n* indicates the number of sperm analyzed. Two‐sided Student's t‐test; error bars, mean ± SEM; *P* values are indicated in the graphs. (E) TEM analysis showing ultrastructural defects in the sperm head of *Wfs1* KO mice. A, acrosome; N, nucleus. Quantification is shown on the right. Scale bars, 200 nm. N = 3 biologically independent mice; *n* indicates the number of sperm analyzed. Two‐sided Student's t‐test; error bars, mean ± SEM; *P* values are indicated in the graphs. (F) TEM analysis of ultrastructural defects in the sperm neck region of *Wfs1* KO mice. Representative images are shown on the left, with schematic diagrams and corresponding quantification shown on the right. Scale bar: 200 nm. N = 3 biologically independent mice; *n* indicates the number of sperm analyzed. Two‐sided Student's t‐test; error bars, mean ± SEM; *P* values are indicated in the graphs. Bp, basal plate; Sc, segmented column; M, mitochondria; N, nucleus; Ax, axoneme; Cap, capitulum. (G) TEM analysis showing disorganized mitochondrial structures in the sperm midpiece of *Wfs1* KO mice. A schematic diagram comparing mitochondrial organization in WT and KO sperm is shown below. Mito, mitochondria; FS, fibrous sheath; Ax, axoneme. Scale bars, 200 nm.

### 3.5 Deficiency of WFS1 Impairs PIAS4‐Associated SUMOylation and Promotes Ubiquitin‐Mediated Degradation of Key Spermatogenesis‐Related Proteins

To elucidate the molecular mechanisms by which WFS1 contributes to male fertility, we performed proteomic analysis of testes from adult WT and *Wfs1* KO mice (Figure 6A), which identified 323 differentially expressed proteins (Figure 6B). Importantly, gene ontology (GO) enrichment analysis further revealed that the majority of downregulated proteins were involved in spermatogenesis‐related processes, including meiotic I cell‐cycle progression, microtubule organization, sperm flagellar motility, and fertilization (Figure 6C). Notably, downregulated proteins were also enriched in pathways related to endoplasmic reticulum (Figure 6G) and mitochondrial functions (Figure 6H). Moreover, several proapoptotic proteins were found to be upregulated (Figure 6D).

**FIGURE 6:**
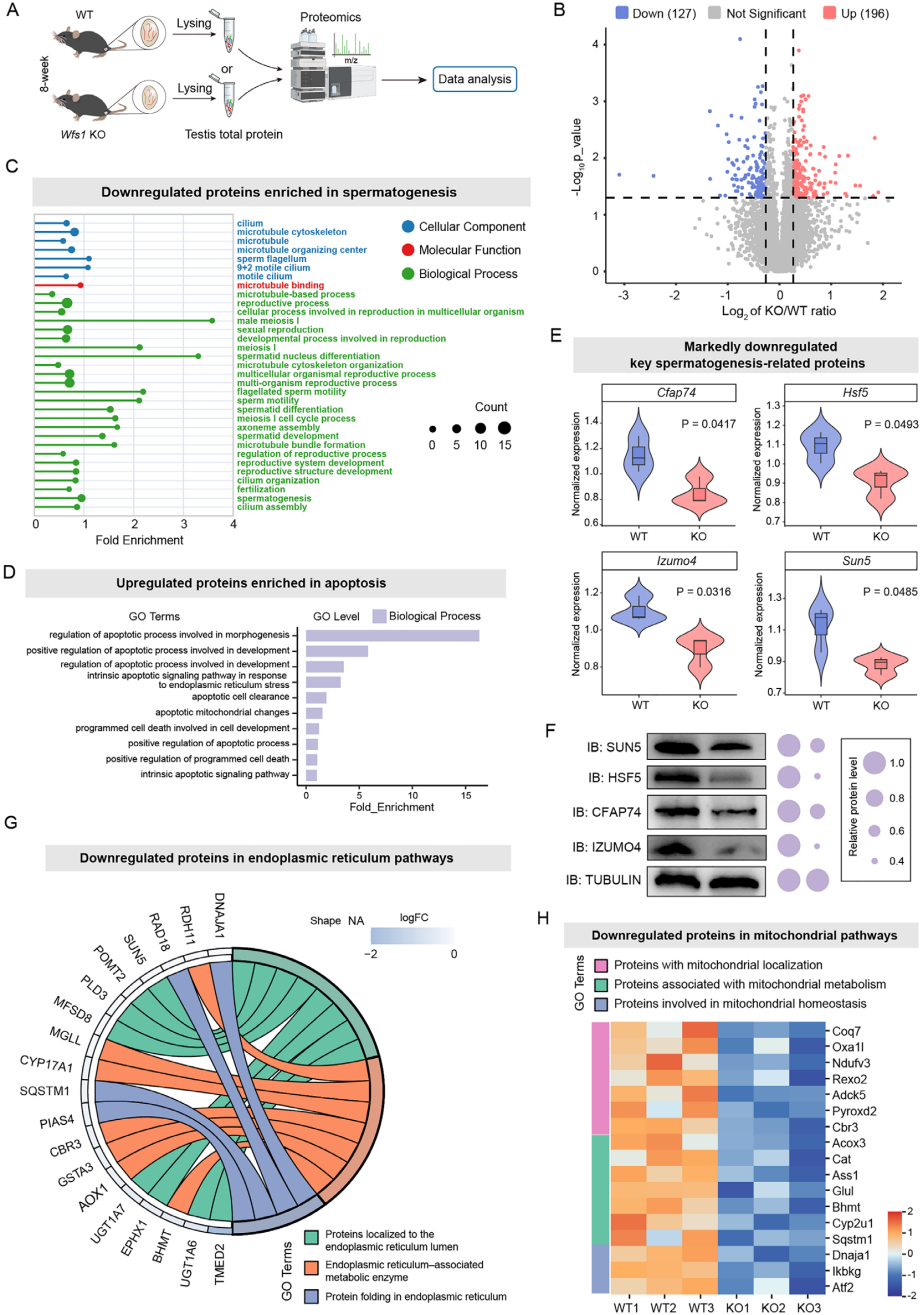
Proteomic analysis reveals dysregulation of spermatogenesis pathways in *Wfs1* KO mice. (A) Schematic diagram illustrating the proteomic workflow, in which total testicular proteins were extracted from three biologically independent WT and *Wfs1* KO mice for proteomic analysis. (B) Volcano plot showing significantly downregulated (blue) and upregulated (red) proteins in *Wfs1* KO versus WT testes. (C) GO analysis showing the majority of downregulated proteins were involved in spermatogenesis‐related processes in biological processes (green), cellular components (blue), and molecular functions (red). Dot size indicates the number of proteins annotated to each term. (D) GO analysis of upregulated proteins showing significant enrichment in apoptosis‐related biological processes. (E) Violin plots showing significant reductions in key spermatogenesis‐related proteins in *Wfs1* KO testes (N = 3 biologically independent WT and KO mice; two‐tailed Student's t‐test). (F) Western blot analysis validating the downregulation of key spermatogenesis‐related proteins in *Wfs1* KO testes. Dot size represents the relative protein levels. (G) Connection network of downregulated proteins participating in endoplasmic reticulum–related functions in *Wfs1* KO mice. (H) Heatmap showing differential expression of proteins involved in mitochondrial pathways between WT and *Wfs1* KO mice.

Among these, we focused on four proteins—SUN5, HSF5, CFAP74, and IZUMO4—that exhibited the most pronounced downregulation, as loss of these proteins in mice has been reported to severely impair male fertility [18, 19, 20, 21, 22, 23] (Figure 6E). Notably, SUN5 is a core component of the HTCA at the sperm neck [24]; HSF5 plays an essential role in sex chromosome synapsis during meiosis [25, 26]; CFAP74 is a critical structural protein of the sperm flagellum [18]; and IZUMO4 is required for sperm–oocyte fusion [19]. Subsequently, Western blot (Figure 6F) and immunofluorescence analyses confirmed that the expression levels of these proteins were significantly reduced in *Wfs1* KO testes (Figure S9B–E). Furthermore, we examined the expression of these proteins in epididymal sperm and found that they were also significantly reduced in *Wfs1* KO sperm (Figure S10A). To confirm that the decreased expression of SUN5, HSF5, CFAP74, and IZUMO4 was caused by WFS1 deficiency rather than germ cell loss, we further analyzed *Wfs1* heterozygous (Het) mice, which exhibited normal seminiferous tubule composition (Figure S10B). Western blot analysis showed that the abundance of these proteins were mildly decreased in Het testes compared with WT testes (Figure S10C).

Ubiquitin‐mediated proteasomal degradation represents a major mechanism regulating protein turnover [27, 28]. As expected, we observed a marked increase in total ubiquitination levels in *Wfs1* KO testes (Figure 7A). Ubiquitin can form polyubiquitin chains through seven lysine residues, with K48 and K63 linkages being the most common [29]. K48 linkages typically mediate proteasomal degradation, whereas K63 linkages is primarily involved in regulating protein activity and localization [30, 31]. Correspondingly, K48‐linked ubiquitination was significantly elevated in KO mouse testes, while K63‐linked ubiquitination remained largely unchanged (Figure 7B). Moreover, K48‐linked ubiquitination of SUN5, HSF5, CFAP74, and IZUMO4 was also significantly increased in KO mice compared to WT controls, accompanied by reduced expression of these proteins (Figure 7C). However, the mechanism by which WFS1 regulates protein ubiquitination remains unclear. Intriguingly, testicular proteomic analysis identified a significant downregulation of the E3‐SUMO ligase PIAS4 in *Wfs1* KO mice (Figure 7D) [32]. We further confirmed an interaction between WFS1 and PIAS4 by co‐immunoprecipitation (Figure 7E), speculating that WFS1 is involved in SUMOylation modification.

**FIGURE 7:**
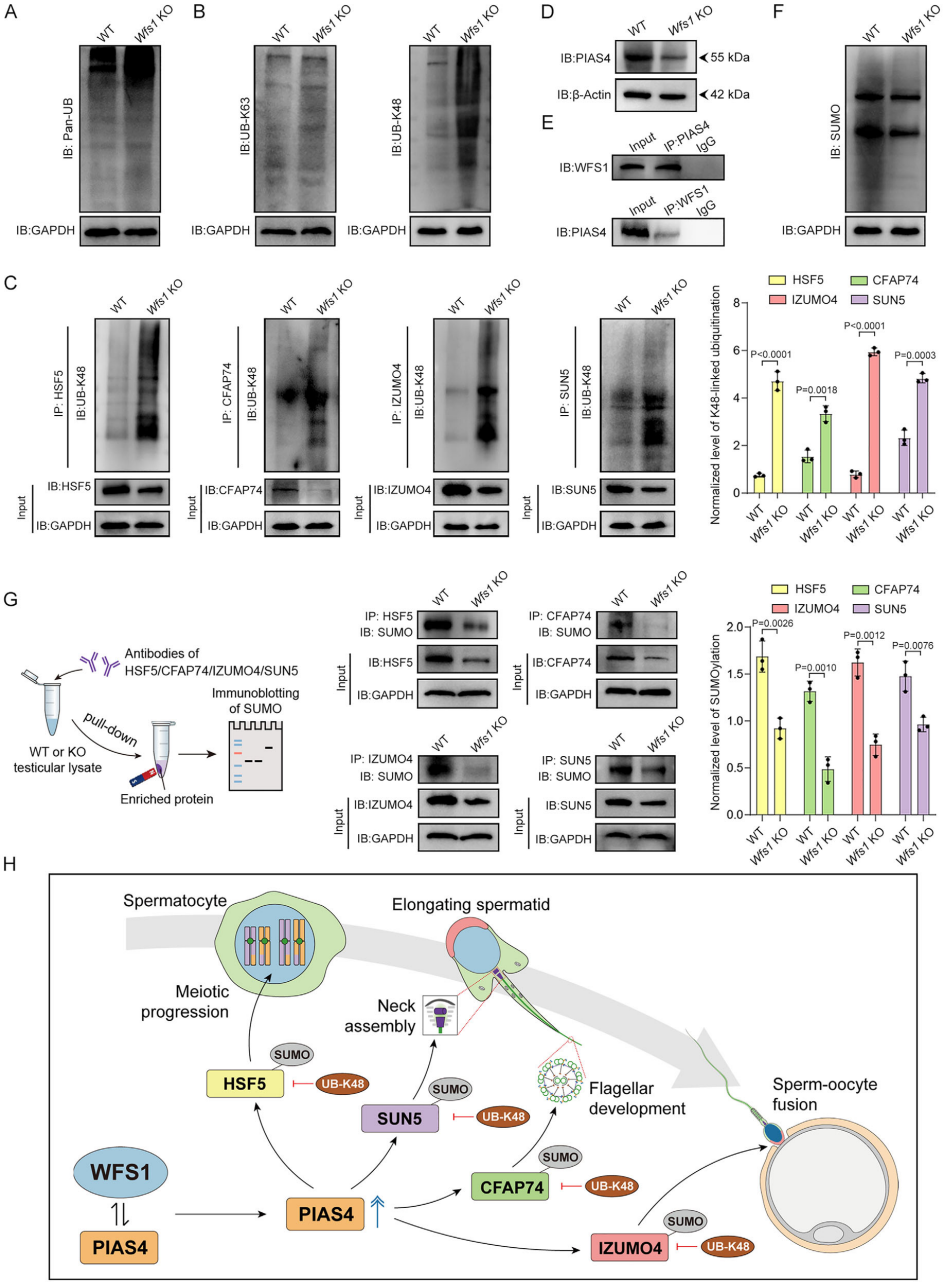
WFS1 regulates spermatogenesis by promoting PIAS4‐dependent SUMOylation, thereby competitively inhibiting K48‐linked ubiquitin‐mediated degradation of key spermatogenesis‐related proteins. (A) Global ubiquitination levels are substantially increased in *Wfs1* KO testes. (B) Further analysis shows a significant elevation of K48‐linked ubiquitination, whereas K63‐linked ubiquitination remains largely unchanged. (C) Co‐immunoprecipitation (Co‐IP) and ubiquitination assays show increased K48‐linked ubiquitination of SUN5, HSF5, CFAP74, and IZUMO4 in *Wfs1* KO testes. Three independent experiments were performed. Two‐sided Student's t‐test; error bars, mean ± SEM; *P* values are indicated in the graphs. (D) Western blot analysis shows downregulation of the E3‐SUMO ligase PIAS4 in *Wfs1* KO testes. (E) Co‐IP assays confirm an interaction between WFS1 and PIAS4 in mouse testes. (F) Western blot analysis reveals a marked decrease in total SUMOylation levels in *Wfs1* KO testes compared to WT controls. (G) Targeted SUMOylation assays demonstrate reduced SUMOylation of SUN5, HSF5, CFAP74, and IZUMO4 in *Wfs1* KO testes. Three independent experiments were performed. Two‐sided Student's t‐test; error bars, mean ± SEM; *P* values are indicated in the graphs. (H) Schematic model illustrating the proposed mechanism: WFS1 interacts with and stabilizes PIAS4 to maintain proper SUMOylation levels and competitively inhibits K48‐linked ubiquitin‐mediated degradation of spermatogenesis‐associated proteins, including HSF5 (essential for chromosomal synapsis), SUN5 (critical for sperm neck formation), CFAP74 (required for flagellar assembly), and IZUMO4 (involved in sperm–oocyte fusion).

Given that post‐translational modifications by ubiquitin and SUMO are key regulators of protein stability and are often functionally antagonistic, disruption of their balance is likely to alter the expression of intracellular proteins [33]. We therefore examined the global SUMOylation status in WT and KO testes. Strikingly, overall SUMOylation levels were markedly decreased (Figure 7F), and further targeted analysis revealed that SUMOylation of these key spermatogenesis‐associated proteins was also significantly reduced in *Wfs1* KO mice (Figure 7G). Taken together, these findings suggested that WFS1 deficiency may lead to downregulation of the E3‐SUMO ligase PIAS4, resulting in reduced SUMOylation and a concomitant increase in K48‐linked ubiquitination of key spermatogenesis‐associated proteins, thereby promoting their proteasomal degradation (Figure 7H).

### 3.6 ICSI Outcomes in Patients and Mice with WFS1 Deficiency

During ICSI procedures, spermatozoa with relatively normal morphology and motility were selected for injection. In this study, ICSI procedures were carried out for two male patients (P219 and P68) carrying *WFS1* variants (Table 3), with informed consent obtained prior to treatment. For patient 219, four metaphase II (MII) oocytes from his partner were injected, resulting in a fertilization rate of 57.14%. Following extended culture, four blastocysts were formed, and two blastocysts were transferred, but no clinical pregnancy was achieved. For patient 68, two oocytes were retrieved from his partner, both of which reached maturity and were successfully fertilized following in ICSI treatment. After extended culture, two day‐3 embryos developed to the blastocyst stage, and the subsequent transfer of these blastocysts resulted in an ongoing pregnancy.

**TABLE 3:** ICSI outcomes in patients with variants in *WFS1*.

	Patient	**P68**	**P219**
**Subjects**	Variant	***WFS1 ^V606G / L734F^***	***WFS1^R772H^***
	Male age (years)	34	37
	Female age (years)	36	38
	Length of primary infertility history (years)	15	5
**Basal hormones**	FSH (IU/L)	7.8	6.5
	LH (IU/L)	5.5	2.4
	E2 (pg/mL)	35	31.8
	Prog (ng/mL)	0.67	0.42
	E2 level on the trigger day (pg/ml)	1961	2771
	No. of follicles ≥ 14 mm on the trigger day	5	9
	No. of follicles ≥ 18 mm on the trigger day	1	3
	No. of oocytes retrieved	2	12
**ICSI progress**	No. of MII oocytes	2	7
	Fertilization rate (%)	100 (2/2)	57.14 (4/7)
	Cleavage rate (%)	100 (2/2)	100 (4/4)
	Available D3 embryos	2	4
**Clinical outcomes**	No. of embryos transferred	2 blastocysts	2 blastocysts
	Implantation rate (%)	100 (2/2)	100 (2/2)
	Clinical pregnancy	Yes	No
	No. of live birth	ongoing	−

E2: estradiol; MII: Metaphase II; ‐, not applicable.

Notably, KO male mice exhibited markedly impaired IVF outcomes (Figure 8A). The pronuclear formation was observed in the majority of embryos from the WT group, whereas the fertilization rate was significantly reduced in the KO group. The compromised fertilization observed in KO mice may be attributed to decreased expression of IZUMO4, a protein implicated in sperm–oocyte adhesion and/or fusion with the oolemma [19, 34].

**FIGURE 8:**
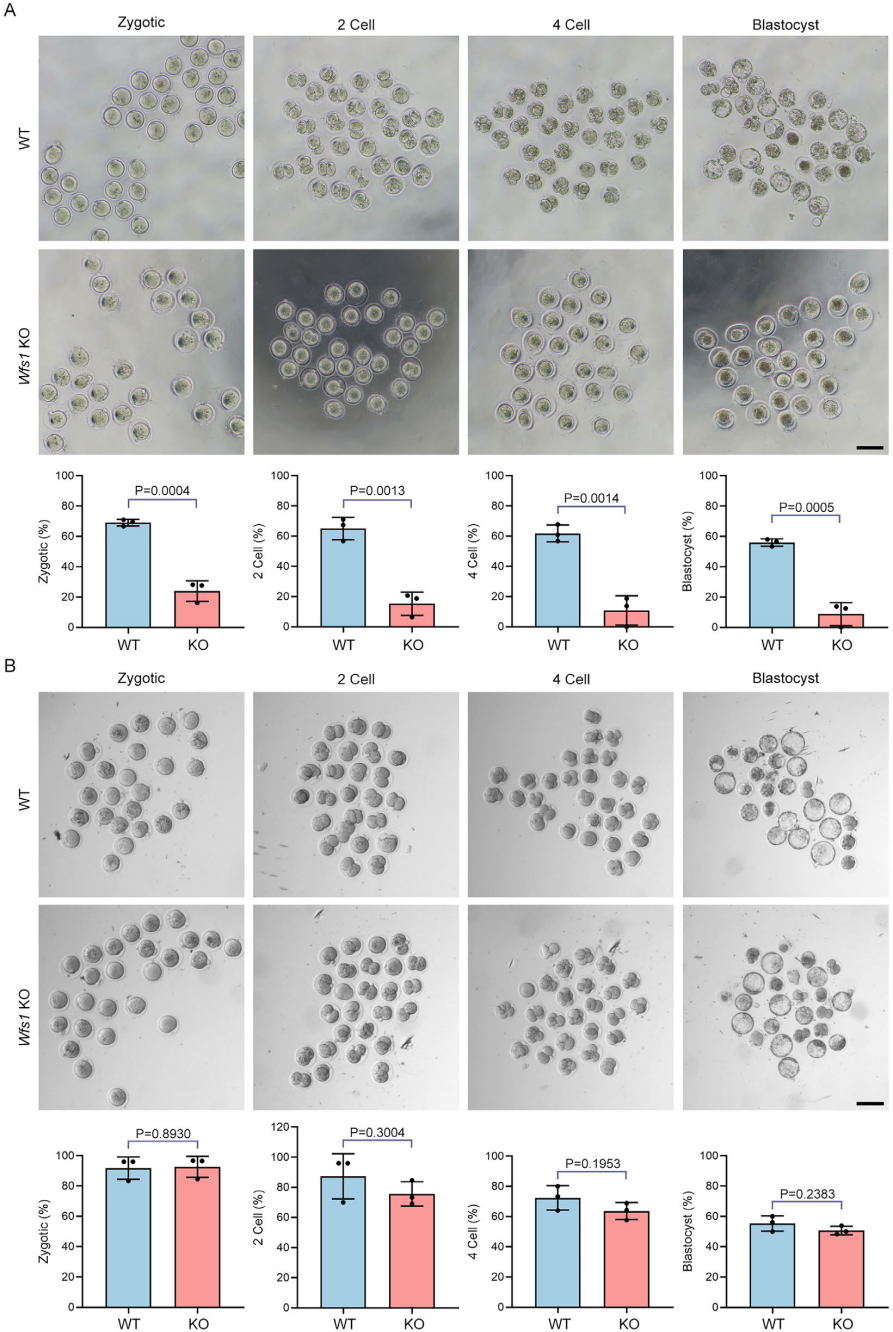
Fertility defects in *Wfs1* KO mice are rescued by ICSI. (A) Representative images of embryos at the zygote, 2‐cell, 4‐cell, and blastocyst stages generated using WT or *Wfs1* KO spermatozoa following in vitro fertilization (IVF). Scale bar, 100 µm. Quantification of developmental outcomes revealed that *Wfs1* KO sperm exhibited significantly reduced rates of progression to the zygote, 2‐cell, 4‐cell, and blastocyst stages compared with WT controls (N = 3 biologically independent WT and KO males; Two‐sided Student's t‐test; error bars, mean ± SEM; *P* values are indicated in the graphs). (B) Representative images of embryos at the zygotic, 2‐cell, 4‐cell, and blastocyst stages generated from WT and *Wfs1* KO spermatozoa following ICSI. Scale bar, 100 µm. Quantification of developmental outcomes showed no significant differences in the formation rates of zygotic, 2‐cell, 4‐cell, or blastocyst stages between WT and KO groups (N  =  3 biologically independent WT and KO males; Two‐sided Student's t‐test; *P* values are indicated in the graphs; error bars, mean ± SEM).

Because chromatin condensation defects were less prevalent in *Wfs1* KO mice than in patients, we further investigated whether *Wfs1* deficient sperm could achieve fertilization and support embryonic development after ICSI. Following injection, most *Wfs1* KO spermatozoa successfully fertilized oocytes and supported embryonic development through the blastocyst stage (Figure 8B). Collectively, these findings indicated that the impact of WFS1 deficiency on fertilization and early embryo development may depend on the severity of sperm chromatin condensation defects. Spermatozoa with less severe chromatin decondensation defects may retain sufficient developmental competence to achieve fertilization and support early embryo development following ICSI.

## 4 Discussion

WFS1 encodes wolframin, an endoplasmic reticulum membrane glycoprotein that plays a critical role in maintaining endoplasmic reticulum homeostasis, calcium signaling, and mitochondrial function [35]. Notably, WFS1 contains three major functional domains, including the WCOB domain, WC‐rich domain, and Wolframin domain. The WCOB domain functions as a protein–protein interaction module that mediates intra‐ and intermolecular interactions and contributes to the interaction of wolframin with partner proteins [36, 37, 38]. Furthermore, the WC‐rich domain may contribute to disulfide bond‐mediated cross‐linking, which is implicated in Ca^2^^+^ store regulation [38, 39]. Finally, the Wolframin domain, located in the central region of the protein, consists of nine transmembrane helices [38, 40, 41, 42]. Despite extensive investigation, the precise role of the Wolframin domain in regulating Ca^2^^+^ flux across the ER membrane remains controversial [8, 38, 43, 44, 45]. Further studies are required to elucidate the precise function of this domain. Pathogenic variants in *WFS1* are well known to cause Wolfram syndrome, a rare autosomal recessive neurodegenerative disorder characterized by diabetes mellitus, optic atrophy, and neurological manifestations [46]. Beyond these classical clinical features, including optic atrophy and diabetes mellitus, previous studies have shown that some Wolfram syndrome patients also exhibit impaired endocrine testicular function and reproductive capacity, such as primary gonadal atrophy, oligozoospermia, and azoospermia [10]. Moreover, a previous *Wfs1*‐deficient mouse model reported reduced male fertility [16]. However, the underlying mechanisms by which WFS1 deficiency causes infertility in humans and mice remain unclear.

In our study, we identified biallelic pathogenic variants in *WFS1* in three unrelated infertile men with oligoasthenoteratozoospermia through WES. The identified missense variants, including c.763G>A and c.1817T>G within the Wolframin domain, c.2122C>T and c.2200C>T within the WC‐rich domain, and c.2315G>A within the WCOB domain, were predicted by three‐dimensional protein structural modeling to impair protein stability. Consistently, transfection of WT‐*WFS1* and mutant‐*WFS1* expression plasmids into cells demonstrated that these missense variants resulted in reduced WFS1 abundance. Clinically, these affected individuals exhibited severe sperm abnormalities, including head malformations, sperm decapitation defects, and mitochondrial disorganization. In addition, we generated *Wfs1* KO mice and found that sperm abnormalities in KO mice were similar to those observed in patients carrying biallelic *WFS1* variants. Furthermore, the KO mice also displayed increased germ cell shedding into the lumen and interstitium of the testes, defective sex chromosome synapsis during meiosis, and ultimately complete infertility. Notably, the phenotype observed in our *Wfs1* KO mice was more severe than the subfertility previously reported in the *Wfs1* deficient mouse model established by Noormets et al. [16]. In their study, *Wfs1* KO males exhibited reduced fertility, which was attributed primarily to abnormal sperm morphology and decreased numbers of spermatogenic cells. In contrast, our *Wfs1* KO males were completely infertile, exhibiting severe sperm morphological abnormalities, reduced sperm numbers, and defective meiotic sex chromosome synapsis. We noted that, in their model, the mutant allele still expresses an N‐terminal WFS1 (amino acids 1–360)–LacZ fusion protein, whereas our exon 3 deletion introduces a frameshift and premature stop codon, which is likely to result in a more complete loss of WFS1 function. In addition, their mice were generated on a W4/129S6 genetic background, whereas our model was established on a pure C57BL/6 background. Differences in genetic background are known to influence phenotypic outcomes in genetically engineered mouse models and may contribute to the discrepancy in phenotypic severity between the two studies [47]. Collectively, these observations suggested that WFS1 may play a more critical role in spermatogenesis and male fertility.

Mechanistically, our findings revealed that WFS1 interacts with PIAS4 to promote the SUMOylation of key spermatogenesis‐associated proteins (SUN5, HSF5, CFAP74, and IZUMO4), thereby competitively inhibiting their ubiquitin‐mediated degradation during spermatogenesis. To further investigate potential shared modification sites between SUMOylation and ubiquitination, we performed in silico prediction analyses using GPS‐SUMO 2.0 and GPS‐Uber, two widely used and validated tools for predicting SUMOylation and ubiquitination sites, respectively [48, 49]. Specifically, SUN5, HSF5, and CFAP74 each contained multiple overlapping candidate sites, and IZUMO4 contained a single overlapping site. Among the predicted sites, Lys76 of IZUMO4 showed the highest prediction score. We therefore generated WT and K76R mutant IZUMO4 constructs to determine whether this mutation affects the SUMOylation and K48‐linked ubiquitination levels of IZUMO4. Co‐IP showed that the K76R mutation reduced both SUMOylation and K48‐linked ubiquitination of IZUMO4, whereas PIAS4 overexpression enhanced SUMOylation and suppressed K48‐linked ubiquitination of WT IZUMO4 but failed to exert these effects on the K76R mutant (Figure S11A–C). These results suggested that SUMOylation and K48‐linked ubiquitination of IZUMO4 may occur at a shared lysine residue and provided experimental support for a potential competitive relationship between these two modifications. Certainly, future studies using comprehensive SUMOylome and ubiquitinome analyses will be required to precisely identify the modification sites of these proteins.

It has been reported that the clinical manifestations of Wolfram syndrome exhibit incomplete penetrance [50]. Accordingly, we have performed a genotype–phenotype correlation analysis of previously reported *WFS1* variants based on the OMIM database and published literature [41]. Variants causing complete loss of WFS1, including frameshift, nonsense, and splicing variants, are predominantly associated with classic Wolfram syndrome involving multiple systemic manifestations. In contrast, patients carrying missense variants generally exhibit milder phenotypes, often presenting with isolated clinical features, such as diabetes mellitus or hearing impairment, rather than the full spectrum of Wolfram syndrome [51, 52, 53]. In addition to the classical features, previous studies have shown that some Wolfram syndrome patients also exhibit impaired endocrine testicular function and reproductive capacity, such as primary gonadal atrophy, oligozoospermia, and azoospermia [10]. In our study, we identified three infertile patients with oligoasthenoteratozoospermia carrying biallelic deleterious *WFS1* variants. Although our mechanistic studies in mouse models demonstrated that WFS1 deficiency reduces the abundance of several key spermatogenesis‐associated proteins, including HSF5, CFAP74, SUN5, and IZUMO4, we cannot exclude the possibility that subclinical metabolic or neurological abnormalities may also contribute to the reproductive phenotype, based on the evidence from the previous Wolfram syndrome patients [5, 10, 11, 12, 13]. Furthermore, the germ cell shedding and detachment into the lumen or interstitial space observed in our *Wfs1* KO testes cannot be fully explained by the reduced abundance of these spermatogenesis‐associated proteins alone and may be associated with impaired Sertoli cell function, as Sertoli cells are essential for germ cell adhesion and maintenance of blood–testis barrier integrity. Importantly, Sertoli cell function is regulated by endocrine signals from the hypothalamic–pituitary–gonadal axis, particularly FSH and testosterone. Consistently, previous studies of Wolfram syndrome patients have reported abnormalities in Sertoli cell function as well as alterations in FSH and testosterone levels [10]. Unfortunately, two of the three patients declined examinations for classical Wolfram syndrome features, we thus cannot determine whether their infertility represents isolated male infertility associated with *WFS1* variants or an incomplete manifestation of Wolfram syndrome. However, patient 68 presented with diabetes, and previous studies have documented reproductive abnormalities in males with Wolfram syndrome [5, 10, 11, 12, 13]. Therefore, we speculated that, in addition to a testis‐intrinsic spermatogenic defect caused by WFS1 deficiency, *WFS1* variant‐associated male infertility may also represent an incomplete manifestation of Wolfram syndrome, with potential contributions from subclinical metabolic or neurological abnormalities.

Male infertility is a common disorder with a prevalence of approximately 7% in human populations [54]. In our study, we identified three infertile patients carrying biallelic *WFS1* variants among 647 infertile patients, indicating that the frequency of infertile men carrying biallelic *WFS1* variants is approximately 0.46% (3/647), which is substantially lower than the prevalence of male infertility. It is reasonable for a recessive pathogenic allele associated with male infertility to have an allele frequency of approximately 1% [55]. Notably, the five *WFS1* variants identified in our study are either extremely rare or absent in population databases (Table 1), with the highest allele frequency being only 0.000087, which is also far below 1%. Furthermore, among an additional 176 newly recruited infertile patients, we did not identify any individuals carrying biallelic *WFS1* variants predicted to be pathogenic. Therefore, compared with the common genetic causes of male infertility, such as Klinefelter syndrome (47, XXY), which accounts for approximately 3%–4% of infertile men [56], and Y chromosome AZF microdeletions, which occur in approximately 3.31% of infertile men [57], *WFS1* variants are likely to represent a relatively rare genetic cause of male infertility.

Beyond WFS1, other members of the wolframin family may also participate in reproductive processes. For instance, emerging evidence indicates that WFS2 (CISD2), another endoplasmic reticulum–mitochondrial tethering protein, plays an essential role in calcium homeostasis and cellular energy balance [58, 59, 60, 61, 62]. Specifically, *Cisd2*‐KO mice have been reported to exhibit a reduction in testicular interstitial cells and abnormal androgen metabolism, ultimately leading to premature focal testicular atrophy [63, 64, 65]. In addition, decreased numbers of Sertoli cells within seminiferous tubules and reduced sperm counts in the epididymis were also observed [63]. Intriguingly, we detected a downregulation of WFS2 in the proteomic analysis of testes from *Wfs1* KO mice. Moreover, STRING analysis also predicted a potential interaction between WFS1 and WFS2, suggesting that they may interact during spermatogenesis. Nevertheless, their potential cooperation within reproduction remains largely unexplored and warrants further investigation.

In summary, our study demonstrated that WFS1 plays a key role in spermatogenesis and fertilization in both humans and mice, expanded the spectrum of *WFS1* variants associated with male infertility, and clarified that WFS1 deficiency‐associated infertility may result from primary testicular defects, with possible contributions from subclinical metabolic or neurological abnormalities. These findings established WFS1 deficiency as a genetic cause of male infertility and provide novel insights with potential implications for diagnosis and clinical management.

## Author Contributions

**Ying Shen** designed and supervised the study. **Yihong Yang** and **Guicheng Zhao** collected semen and blood samples and performed clinical evaluations of the participants. **Yunchuan Tian** and **Xinyao Tang** conducted western blot, immunofluorescence staining, and data analysis. **Gan Shen** and **Chuan Jiang** performed cytometric analysis and cell sorting. **Xiang Wang** and **Tiechao Ruan** generated schematic diagrams. **Jincheng Zhang** and **Xinyue Cui** performed Sanger sequencing. **Yunchuan Tian**, **Shikun Zhao**, **Liangchai Zhuo**, and **Jun Ma** contributed to phenotypic analysis. **Tingting Lin** provided the animal models. **Yunchuan Tian** and **Xinyao Tang** drafted the manuscript. **Ying Shen** critically reviewed the final version of the manuscript. All authors contributed to revising and reviewing the manuscript.

## Funding

This work was financed by the National Natural Science Foundation of China (82471650).

## Conflicts of Interest

The authors declare no conflicts of interest.

## Supporting information




**Supporting File 1**: advs78060‐sup‐0001‐SuppMat.png.

**Supporting File 2**: advs78060‐sup‐0002‐SuppMat.png.

**Supporting File 3**: advs78060‐sup‐0003‐SuppMat.png.

**Supporting File 4**: advs78060‐sup‐0004‐SuppMat.png.

**Supporting File 5**: advs78060‐sup‐0005‐SuppMat.png.

**Supporting File 6**: advs78060‐sup‐0006‐SuppMat.png.

**Supporting File 7**: advs78060‐sup‐0007‐SuppMat.png.

**Supporting File 8**: advs78060‐sup‐0008‐SuppMat.png.

**Supporting File 9**: advs78060‐sup‐0009‐SuppMat.png.

**Supporting File 10**: advs78060‐sup‐0010‐SuppMat.png.

**Supporting file 11**: advs78060‐sup‐0011‐SuppMat.png

**Supporting File 12**: advs78060‐sup‐0012‐TableS2.docx.

**Supporting File 13**: advs78060‐sup‐0013‐TableS2.docx.

**Supporting File 14**: advs78060‐sup‐0014‐TableS3.docx.

## Data Availability

The data that support the findings of this study are available from the corresponding author upon reasonable request.
