# Supplementary material for: WFS1 Deficiency Impairs PIAS4‐Associated SUMOylation and Increases Ubiquitin‐Mediated Spermatogenesis‐Related Protein Degradation Leading to Testicular Male Infertility
Source: Adv Sci (Weinh). 2026 Oct 4:e78060. Online ahead of print. doi: 10.1002/advs.78060 (PMC13634479; doi:10.1002/advs.78060)
Supplement: Supplementary file 1 [file ADVS-9999-e78060-s003.docx]

**Supplementary Table S3. Comprehensive list of antibodies employed in this study.**

| **Antibody or dye (application and dilution ratio)** | **Source** | **Identifier** |
| --- | --- | --- |
| Anti-PNA (IF, 1:50) | Invitrogen | L-32460 |
| Anti-α-tubulin (IF, 1:200) | Abcam | AB7291 |
| Alexa Fluor 488 (IF, 1:500) | Thermo Fisher | A11008 |
| Alexa Fluor 594 (IF, 1:500) | Thermo Fisher | A11005 |
| Anti-GAPDH (WB, 1:1000) | Huabio | ET1601-4 |
| Anti-WFS1 (IF, 1:50; WB, 1:1000) | Proteintech | 26995-1-AP |
| Anti-TOM20 (IF, 1:50) | Proteintech | 11802-1-AP |
| Anti-HSF5 (IF, 1:100; WB, 1:1000; IP) | Atlas antibodies | HPA063613 |
| Anti-CFAP74 (IF, 1:50; WB, 1:500; IP) | Bioss | BS-13802R |
| Anti-SUN5 (IF, 1:50; WB, 1:400; IP) | Proteintech | 17495-1-AP |
| Anti-IZUMO4 (IF, 1:50; WB, 1:300; IP) | Sigma-Aldrich | HPA048496 |
| Anti-α-tubulin (IF, 1:50; WB, 1:1000) | Huabio | M1501-1 |
| Anti-α-tubulin (IF, 1:50) | Santa Cruz Biotechnology | sc-32293 |
| Anti-β-Actin (WB, 1:1000) | PTMab | PTM5436 |
| Anti-Flag (WB, 1:3000) | Proteintech | 66008-4-Ig |
| Anti-HA (WB, 1:3500) | Proteintech | 51064-2-AP |
| Anti-Ubiquitin (WB, 1:1000) | Cell signaling technology | 58395 |
| Anti-HORMAD1 (IF, 1:100) | Atlas antibodies | HPA028346 |
| Anti-γH2AX (IF, 1:100) | Abcam | AB26350 |
| Anti-MLH3 (IF, 1:100) | This paper | Gift from Prof. Mengcheng Luo |
| Anti-SYCP1 (IF, 1:50) | Abcam | AB15090 |
| Anti-SYCP3 (IF, 1:20) | Santa Cruz Biotechnology | sc-74569 |
| Anti-SYCP3 (IF, 1:100) | Abcam | AB97672 |
| Anti-PIASy (PIAS4) (WB, 1:1000; IP) | Santa Cruz Biotechnology | sc-166706 |
| Anti-Ubiquitin (linkage-specific K48) (WB, 1:1000) | PTMab | PTM7205 |
| Anti-Ubiquitin (linkage-specific K63) (WB, 1:1000) | PTMab | PTM7228 |
| Anti-SUMO1/2/3 (WB, 1:1000) | PTMab | PTM1109 |
| Anti-c-Kit (IF, 1:50) | Proteintech | 18696-1-AP |
| Anti-SOX9 (IF, 1:100) | Abcam | AB185966 |
| Anti-Collagen IV (IF, 1:100) | Bioss | BS-4595R |
| Anti-ZO-1 (IF, 1:50) | Bioss | BS-1329R |
| Goat Anti-Rabbit IgG (WB, 1:5000) | Jackson | 111-035-003 |
| Goat Anti-Mouse IgG (WB, 1:5000) | Jackson | 115-035-003 |
| IPKine™ HRP, Goat Anti-Mouse IgG (WB, 1:5000) | Abbkine | A25012 |
| IPKine™ HRP, Mouse Anti-Rabbit IgG (WB, 1:5000) | Abbkine | A25022 |
