# Supplementary material for: WFS1 Deficiency Impairs PIAS4‐Associated SUMOylation and Increases Ubiquitin‐Mediated Spermatogenesis‐Related Protein Degradation Leading to Testicular Male Infertility
Source: Adv Sci (Weinh). 2026 Oct 4:e78060. Online ahead of print. doi: 10.1002/advs.78060 (PMC13634479; doi:10.1002/advs.78060)
Supplement: Supplementary file 2 [file ADVS-9999-e78060-s005.docx]

**Supplementary Table S1. Over view of the primer sequences used in this study.**

| **Application of primers** | **Sequences** |
| --- | --- |
| **Sanger sequencing validation** |  |
| c.763G>A | F 5’- TGGAGGCTGGCACTTGGCAAA -3’ |
|  | R 5’- GTAAGACACTCTTTGTTAGGCGCG -3’ |
| c.1817 T>G, c.2200 C>T | F 5’- CTGCTCTATGTCTACCTGCTCTATC -3’ |
|  | R 5’- CTGAGCAGCACGCTCTTGAACTC -3’ |
| c.2122C>T | F 5’- TGTGGTGGGGATGGTGAAGTCC -3’ |
|  | R 5’- GAATGGCATGCCCACGGTAAT -3’ |
| c.2315G>A | F 5’- TGCCATCAACATGCTCCCGTTC -3’ |
|  | R 5’- CATGCAGTTGAGGCAGCTGATG -3’ |
|  |  |
| ***WFS1* plasmid construction** |  |
| *WFS1* | F 5’- GACAAGGGATCCGGGATGGACTCCAACACTGCTCC -3’ |
|  | R 5’- AACGGGCCCTGAATTTCAGGCCGCCGACAGGAAT -3’ |
|  |  |
| **Patient-derived point mutations** |  |
| c.763G>A | F 5’- TCACTAAGAAGTACACCAAGGGCGT -3’ |
|  | R 5’- TGTACTTCTTAGTGATCTCCACAA -3’ |
| c.1817 T>G | F 5’- TGGCGGTCTGTAGTGGGCCCCTGCT -3’ |
|  | R 5’- CCACTACAGACCGCCACGGTGACTG -3’ |
| c.2122C>T | F 5’- GCTTCAAGTACGTCTGCGTGACTGA -3’ |
|  | R 5’- AGACGTACTTGAAGCGGCCGGTCCA -3’ |
| c.2200 C>T | F 5’- TGGATGCGCTGCTTCTACGGCGAGG -3’ |
|  | R 5’- AGCAGCGCATCCAGTCGCCGATGAA -3’ |
| c.2315G>A | F 5’- CAAGAAGTTCGACCACTACAAGTTT -3’ |
|  | R 5’- TGGTCGAACTTCTTGATGTGGCAGG -3’ |
