# Supplementary material for: WFS1 Deficiency Impairs PIAS4‐Associated SUMOylation and Increases Ubiquitin‐Mediated Spermatogenesis‐Related Protein Degradation Leading to Testicular Male Infertility
Source: Adv Sci (Weinh). 2026 Oct 4:e78060. Online ahead of print. doi: 10.1002/advs.78060 (PMC13634479; doi:10.1002/advs.78060)
Supplement: Supplementary file 3 [file ADVS-9999-e78060-s008.docx]

**Supplementary Table S2. Primers for genotyping and sgRNA target sequences.**

| **Application of primers** | **Sequences** | **Product (bp)** |
| --- | --- | --- |
| sgRNA1 for *Wfs1* KO mice | 5’- AGGGAAGACGTCCACTCTTAAGG -3’ |  |
| sgRNA2 for *Wfs1* KO mice | 5’- ACCTTGTTGATCTGTTGATCTGG -3’ |  |
| *Wfs1* KO genotyping-1 | F1 5’- GTGCCACCATGAACACTAAAGC -3’ | WT: 1599 |
|  | R1 5’- AGGGCAACTTGTGGGATTCAG -3’ | KO: 274 |
| *Wfs1* KO genotyping-2 | F2 5’- TGCAACAGGCTTGTTCCCTAAG -3’ | WT: 312 |
|  | R2 5’- TGAGACGGACTACACTGTCCAAC -3’ | KO: 0 |
| *Wfs1***^a^** | F 5’- GTTTCGGAGCAACTTCGCG -3’ | 179 |
|  | R 5’- ACGAGGCGGTTCAATCTTGT -3’ |  |

^a^ Primer sequences for quantitative PCR.
